# High-Contrast Detection of Somatostatin Receptor Subtype-2 for Fluorescence-Guided Surgery

**DOI:** 10.1021/acs.molpharmaceut.2c00583

**Published:** 2022-09-29

**Authors:** Servando Hernandez Vargas, Solmaz AghaAmiri, Sukhen C. Ghosh, Michael P. Luciano, Luis C. Borbon, Po Hien Ear, James R. Howe, Jennifer M. Bailey-Lundberg, Gregory D. Simonek, Daniel M. Halperin, Hop S. Tran Cao, Naruhiko Ikoma, Martin J. Schnermann, Ali Azhdarinia

**Affiliations:** † The Brown Foundation Institute of Molecular Medicine, McGovern Medical School, The University of Texas Health Science Center at Houston, Houston, Texas77054, United States; ‡ Chemical Biology Laboratory, Center for Cancer Research, National Cancer Institute, Frederick, Maryland21702, United States; § Department of Surgery, University of Iowa Carver College of Medicine, Iowa City, Iowa52242, United States; ∥ Department of Anesthesiology, McGovern Medical School, The University of Texas Health Science Center at Houston, Houston, Texas77030, United States; ⊥ Center for Laboratory Animal Medicine and Care, McGovern Medical School, The University of Texas Health Science Center at Houston, Houston, Texas77030, United States; # Department of Gastrointestinal Medical Oncology, The University of Texas MD Anderson Cancer Center, 1515 Holcombe Blvd., Houston, Texas77030, United States; ∇ Department of Surgical Oncology, The University of Texas MD Anderson Cancer Center, 1515 Holcombe Blvd., Houston, Texas77030, United States

**Keywords:** fluorescence-guided surgery, intraoperative imaging, cancer surgery, dual labeling, near-infrared fluorescence imaging, somatostatin receptor

## Abstract

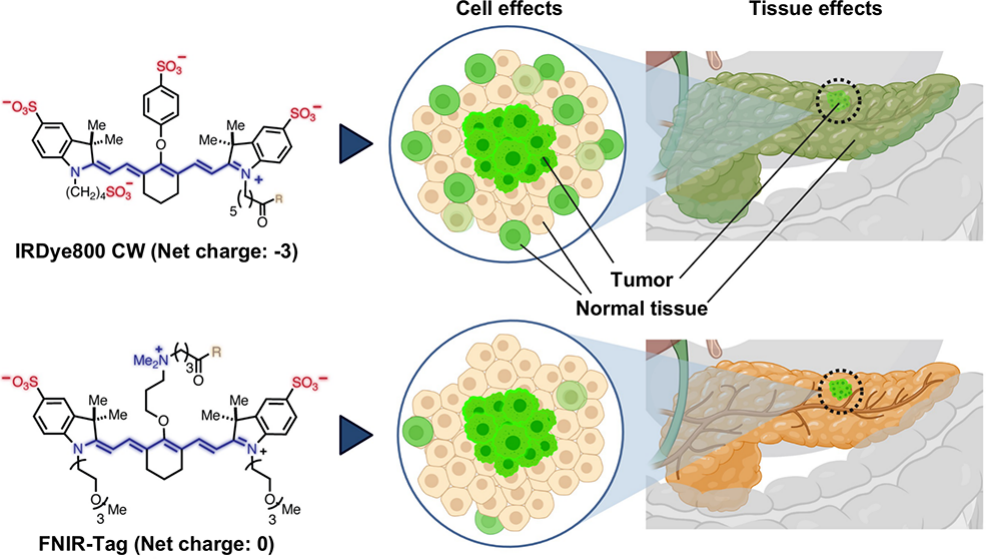

Dye design can influence the ability of fluorescently labeled imaging agents to generate tumor contrast and has become an area of significant interest in the field of fluorescence-guided surgery (FGS). Here, we show that the charge-balanced near-infrared fluorescent (NIRF) dye FNIR-Tag enhances the imaging properties of a fluorescently labeled somatostatin analogue. *In vitro* studies showed that the optimized fluorescent conjugate MMC(FNIR-Tag)-TOC bound primarily *via* somatostatin receptor subtype-2 (SSTR2), whereas its negatively charged counterpart with IRDye 800CW had higher off-target binding. NIRF imaging in cell line- and patient-derived xenograft models revealed markedly higher tumor contrast with MMC(FNIR-Tag)-TOC, which was attributed to increased tumor specificity. *Ex vivo* staining of surgical biospecimens from primary and metastatic tumors, as well as involved lymph nodes, demonstrated binding to human tumors. Finally, in an orthotopic tumor model, a simulated clinical workflow highlighted our unique ability to use standard preoperative nuclear imaging for selecting patients likely to benefit from SSTR2-targeted FGS. Our findings demonstrate the translational potential of MMC(FNIR-Tag)-TOC for intraoperative imaging and suggest broad utility for using FNIR-Tag in fluorescent probe development.

## Introduction

Surgery is the primary treatment option for most solid tumors and can be curative if all cancer cells are removed. Accurate intraoperative detection of tumors is therefore essential and has led to the development of techniques that augment visual identification of cancer in the operating room. Fluorescence-guided surgery (FGS) is a method of enhancing intraoperative visualization of tumors, which may be difficult to discern, often through the use of exogenously administered contrast agents that target tumor tissues. This approach allows for delineation of tumors from normal tissues in real time to enable safer and more effective removal of cancerous lesions.^1^ Increasing evidence suggests that the chemical features of the fluorescent label can strongly influence the *in vivo* imaging properties of bioconjugates, including their specificity and ability to generate contrast.^2,3^ Thus, dye selection is critical and has recently emerged as a key technical element of FGS research.^4−6^

Cyanine dyes that emit fluorescence in the near-infrared (NIR) range (≥700 nm) provide strong depth of penetration and capitalize on the inherently low tissue autofluorescence in this spectral range, making them a preferred class of fluorophore for FGS procedures.^5^ We recently reported a chemically stable C4′-*O*-alkyl charge-balanced cyanine fluorophore, FNIR-Tag, that is highly promising for biomolecule labeling and imaging.^7^ FNIR-Tag conjugates of antibodies or virus-like particles were brighter and had improved tumor targeting and reduced nonspecific (*i.e.*, liver) uptake when compared to their counterparts that used the highly anionic (net charge −3) fluorophore IRDye800 CW (abbreviated as IR800). FNIR-Tag could offer similar benefits with low-molecular-weight compounds (*e.g.*, peptides), which are inherently more sensitive to bioconjugation effects, by reducing nonspecific interactions and altered pharmacokinetics (*e.g.*, excretion rates) caused by labeling with highly charged dyes.^6^

Gastroenteropancreatic neuroendocrine tumors (GEP-NETs) are a heterogeneous group of malignancies with substantially increasing incidence and prevalence.^8^ There are two key elements that make GEP-NETs an ideal environment for evaluating the performance of a low-molecular-weight FNIR-Tag conjugate. First, GEP-NETs rely on surgery as a critical component of patient care but lack technologies^9^ that can identify small, multifocal lesions or involved lymph nodes, both of which are common in this disease process or tumor margins in the operating room.^10−13^ Second, these patients are administered clinically approved radioactive somatostatin analogues for preoperative imaging and surgical planning *via* a somatostatin receptor subtype-2 (SSTR2)-targeted positron emission tomography (PET) scan,^14,15^ thereby establishing the value of the somatostatin analogue/SSTR2 ligand-receptor axis. Since somatostatin analogues can undergo a wide range of bioconjugation reactions without losing binding affinity, we developed a strategy to transform the PET agent 1,4,7,10-tetraazacyclododecane-1,4,7,10-tetraacetic acid-Tyr^3^-octreotide (^68^Ga-DOTA-TOC; ^68^Ga = Gallium-68, a positron-emitting radionuclide; TOC = SSTR2-targeting peptide) from a diagnostic agent into a surgical navigation tool by synthesizing a dual-labeled analogue, ^68^Ga-MMC(IR800)-TOC.^16^ The MMC (multimodality chelator) is a customized cyclen analogue that enables facile conjugation of IR800 while also acting as a “radioactive linker” for noninvasive imaging and *ex vivo* tissue quantitation. Unlike conventional FGS agents, inclusion of the MMC enabled quantitative comparison to ^68^Ga-DOTA-TOC as a benchmark and showed that dye conjugation did not impair SSTR2 binding in cells and animal models.^16,17^ Furthermore, *ex vivo* staining of pancreatic NET (pNET) biospecimens showed high specificity for human SSTR2-expressing tumors and correlated with gold standard histopathology, demonstrating for the first time that a clinical radiotracer could be adapted for FGS.

**Figure 1:**
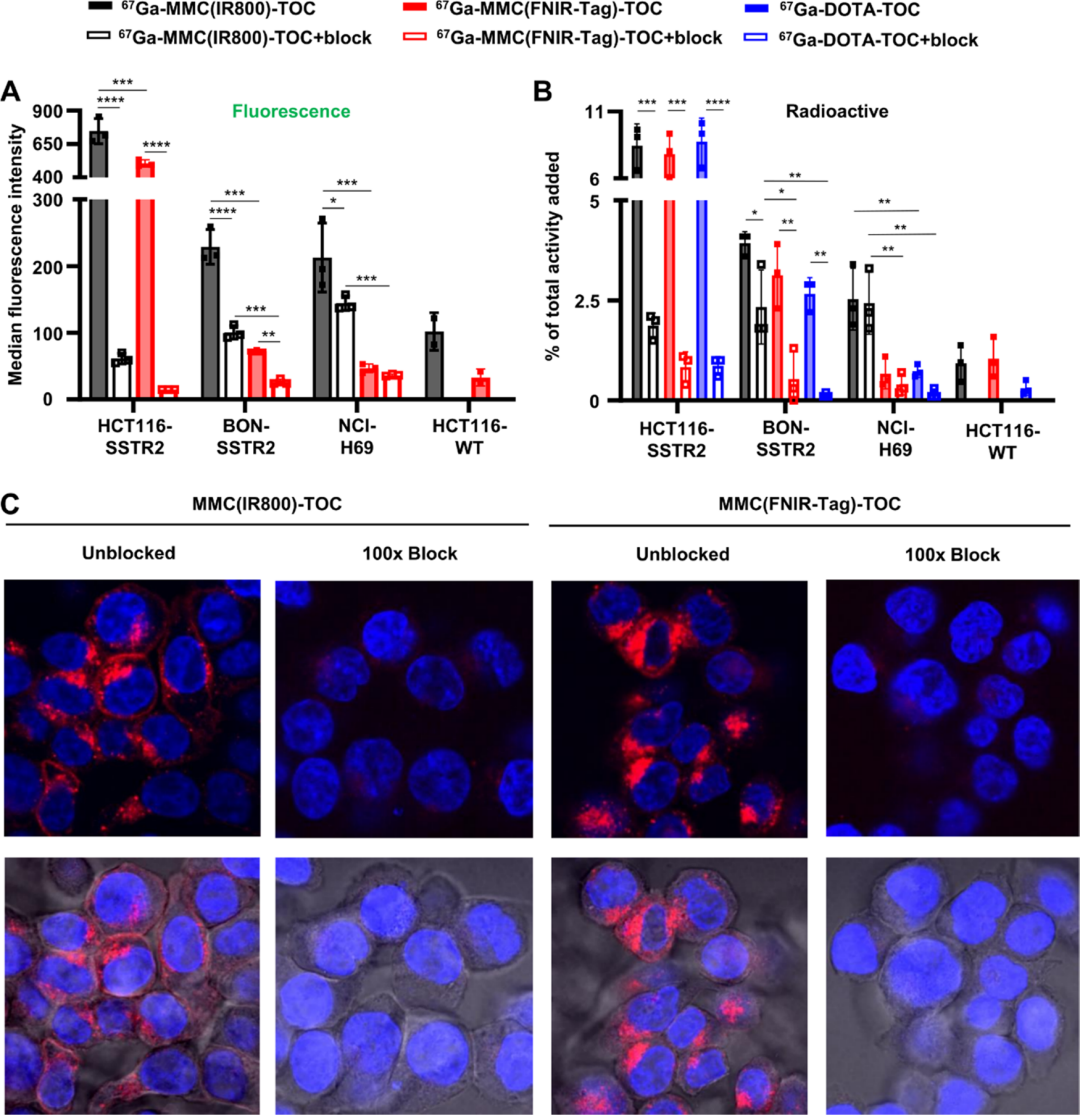
*In vitro* binding of dual-labeled conjugates. Binding of ^67^Ga-MMC(IR800)-TOC and ^67^Ga-MMC(FNIR-Tag)-TOC in cell lines with varying SSTR2 expression (HCT116-SSTR2 ≫ BON–SSTR2 > NCI-H69) as shown by (A) flow cytometry and (B) radioactive uptake. Results are presented as mean ± s.d. (*n* = 3). ^****^P ≤ 0.0001, ^***^P ≤ 0.001, ^**^P ≤ 0.01, and *P < 0.05. A 100-fold excess of octreotide was used as a blocking agent in both experiments, and ^67^Ga-DOTA-TOC was used as a control in radioactive studies. The SSTR2-negative cell line, HCT116-WT, was not blocked. (C) Confocal microscopy images in the presence and absence of blocking. Top row, fluorescent channels only; bottom row, merged bright field and fluorescent channels.

Despite the excellent tumor-targeting properties of the first-generation conjugate, prolonged blood half-life and high background signal in NET-associated tissues (*e.g.*, pancreas, small intestine) prevented clear contrast at clinically desirable time points (3 h post-injection). Thus, we hypothesized that replacing IR800 with an optimized NIR fluorophore would reduce nonspecific interactions, restore the favorable pharmacokinetic profile of somatostatin analogues, and provide meaningful image contrast at time points that are compatible with GEP-NET surgery in a clinical setting. Here, we synthesized a second-generation analogue, MMC(FNIR-Tag)-TOC, with the overall goal of improving tumor specificity and suitability for clinical imaging (Figure 1). Using multiple model systems, we show that MMC(FNIR-Tag)-TOC consistently outperformed its IR800 counterpart at the cellular, tissue, and whole-body levels, suggesting high potential for translational studies in SSTR2-expressing cancers.

## Methods

### Materials and General Methods

All chemicals were purchased from Sigma-Aldrich (Saint Louis, MO) unless otherwise noted. IR800-DBCO was purchased from LI-COR Biosciences (Lincoln, NE). Reversed-phase high-performance liquid chromatography (HPLC) was performed on an analytical Hitachi LaChrom system using a Kinetex C18 column (5 μm, 50 mm × 4.6 mm) (Phenomenex) with a mobile phase of *A* = 0.1% TFA in H_2_O and *B* = 0.1% TFA in CH_3_CN (gradient: 0 min, 10% B; 10 min, 90% B); flow rate, 1 mL/min. Electrospray ionization mass spectra were acquired on a LCQ FLEET instrument (Thermo Scientific). Flash column chromatography was performed using reversed phase (100 Å, 20–40 μm particle size, RediSep Rf Gold Reversed-phase C18Aq).

### Synthesis of FNIR-Tag-DBCO

FNIR-Tag (27 mg, 0.025 mmol) and HATU (19 mg, 0.050 mmol, 2 equiv) were dissolved in dry DMF (1.3 mL) in a 1-dram vial equipped with a magnetic stir bar. DIPEA (13 μL, 0.076 mmol, 3 equiv) was added under argon, and the green solution was stirred for 0.5 h at ambient temperature. DBCO-amine (7.7 mg, 0.028 mmol, 1.1 equiv) dissolved in dry DMF (350 μL) was added under argon, and the reaction was stirred for an additional 0.5 h. The reaction mixture was precipitated in diethyl ether (30 mL) in a 50 mL conical tube, vortexed, and centrifuged for 5 min at 5000 RPM. The pellet was dissolved in 5% acetonitrile/water (10.0 mL) and directly purified by automated reversed-phase flash chromatography (15.5 g C18Aq, 0–50% MeCN/water). The green fractions were combined and lyophilized to afford FNIR-Tag-DBCO (14.1 mg, 42% yield) as a fluffy green powder. ^1^H NMR (500 MHz, MeOD) δ 8.10 (d, *J* = 14.2 Hz, 2H), 7.88 (dd, *J* = 9.9, 1.8 Hz, 4H), 7.64 (d, *J* = 8.0 Hz, 1H), 7.49 (dd, *J* = 6.3, 2.1 Hz, 1H), 7.40–7.29 (m, 7H), 7.24 (d, *J* = 9.0 Hz, 1H), 6.35 (d, *J* = 14.2 Hz, 2H), 5.14 (d, *J* = 14.2 Hz, 1H), 4.37 (t, *J* = 5.2 Hz, 4H), 4.14 (t, *J* = 6.2 Hz, 2H), 3.90 (t, *J* = 5.1 Hz, 4H), 3.75–3.70 (m, 2H), 3.68 (d, *J* = 14.0 Hz, 1H), 3.59 (dd, *J* = 6.0, 3.0 Hz, 5H), 3.52 (dd, *J* = 5.6, 3.4 Hz, 4H), 3.50–3.38 (m, 11H), 3.29 (s, 6H), 3.26 (two overlapping s, 6H), 2.66 (t, *J* = 6.3 Hz, 4H), 2.56–2.46 (m, 3H), 2.23 (t, *J* = 6.9 Hz, 2H), 2.12–2.01 (m, 3H), 1.92 (t, *J* = 6.0 Hz, 2H), 1.75 (s, 6H), 1.72 (s, 6H) ppm. HRMS (Q-TOF) calculated for C_71_H_93_N_5_O_15_S_2_ (M + H)^+^ 1319.6099, observed 1319.6080.

### Synthesis, Spectral, and Physicochemical Characterization of Fluorescent Conjugates

FNIR-Tag-DBCO was conjugated to azido-MMC-TOC according to methods described for MMC(IR800)-TOC.^16^ Briefly, a solution of azido-MMC-TOC (1.5 mg, 0.985 μmol) was mixed with FNIR-Tag-DBCO (1.4 mg, 1.060 μmol) in a mixture of water and DMSO (3:1). After stirring at 37 °C for 6 h and overnight at room temperature in the dark, the product (∼1.6 mg) was purified with an ultrafiltration spin column (2000 Da molecular weight cutoff). Purified MMC(FNIR-Tag)-TOC was identified by analytical HPLC and electrospray mass spectrometry *m*/*z* calculated for C_142_H_193_N_23_O_34_S_4_, 2895.66; found *m*/*z*, 1448.1 (1/2 mass) for [M + H]^+^. Chemical purity of 96.5% was determined by HPLC with a retention time of 6.14 min for the product peak. Absorption curves were collected on a Shimadzu UV-2550 spectrophotometer operated by UVProbe 2.32 software. Fluorescence traces were recorded on a Horiba PTIQuantaMaster-400 fluorometer operated by FelixGX 4.2.2 software, with 5 nm excitation and emission slit widths, 0.1 s integration rate, and enabled emission correction. The Horiba PTIQuantaMaster-400 fluorometer was equipped with an integrating sphere for absolute Φ_F_ measurements. Absolute Φ_F_ measurements were carried out on solutions with absorbance at λ_max_ < 0.1. Calculated Log P (c Log P) values were calculated using ChemDraw Professional v19.1.

### Cell Culture

NCI-H69 (human bronchial NET; ATCC), HCT116-WT (human colorectal carcinoma; SSTR2-), and HCT116-SSTR2 (transfected SSTR2-expressing counterpart) cells were cultured as previously described.^17^ BON–SSTR2 (human pNET; transfected to overexpress SSTR2) were cultured in DMEM medium with 10% (v/v) FBS and 250 μg/mL G418 antibiotic and maintained at 37 °C with 95% humidity/5% CO_2_ atmosphere. HCT116-WT and HCT116-SSTR2 cells were kindly provided by Dr. Buck Rogers (Washington University in St. Louis). BON–SSTR2 cells were courtesy of Dr. Jeffrey Frost (McGovern Medical School, Houston, TX). Routine testing was performed to confirm the absence of mycoplasma in cell lines using the MycoAlert PLUS Mycoplasma Detection Kit (Lonza; Catalog #: LT07-703).

### Animal Models

All animal studies were performed in accordance with the ethical protocols approved by the Institutional Animal Care and Use Committee (IACUC) of the University of Texas Health Science Center at Houston. Athymic female J:Nu nude mice (6–8 weeks old; The Jackson Laboratory) were maintained on regular rodent chow and anesthetized with 1–2% isoflurane when required. For subcutaneous xenografts, cells were prepared in Matrigel (Corning)/PBS (1:1) and injected in the shoulders of mice. For the bilateral HCT116-SSTR2/WT xenograft model, 1.5 and 3 × 10^6^ cells were implanted, respectively. For the NCI-H69 xenograft model, 6 × 10^6^ cells were implanted. Experiments were conducted 3–4 weeks post-implantation when tumor size reached ∼5–10 mm maximum diameter. To enable testing in a more clinically representative setting, an orthotopic pancreatic tumor model was also developed according to published methods.^18,19^ Mice were anesthetized, and a survival surgery was performed under aseptic conditions. The pancreas was then exposed to inject 3 × 10^6^ BON–SSTR2 cells in a Matrigel/PBS mixture (1:1, 50 μL total volume) into the head of the pancreas. Studies were conducted 8 weeks after orthotopic implantation. To further strengthen the rigor of our *in vivo* studies, a bilateral PDX tumor model was prepared using procedures approved by The University of Iowa’s Institutional Animal Care and Use Committee. NOD scid γ mice (stock no: 005557; The Jackson Laboratory) were injected with 1 × 10^6^ NEC913 (SSTR2+) and 7 × 10^5^ NEC1452 (SSTR2-) NEC cells, as previously described, and studies were performed 4 weeks after implantation.^20^ We used the percutaneous retro-orbital technique for intravenous injections and overdose of anesthesia followed by cervical dislocation as the method of euthanasia in terminal studies unless otherwise noted.

### Radiochemistry

Short and longer-lived Ga radionuclides were used based on experimental objectives. Generator-produced ^68^GaCl_3_ (*t*_1/2_ = 68 min) was purchased from the MD Anderson radiopharmacy (Houston, TX) and ^67^Ga-citrate (*t*_1/2_ = 3.3 d) was purchased from Cardinal Health. DOTA-TOC, MMC(IR800)-TOC, and MMC(FNIR-Tag)-TOC (20 nmol per batch) were dissolved in sodium acetate buffer and radiolabeled with ^68^Ga or ^67^Ga using cation exchange chromatography as previously described.^17^ Following purification with a Sep-Pak Light C18 (Waters) cartridge, radiochemical purities were determined by radio-HPLC using a dual scan-RAM (LabLogic) and found to be ≥95% (Supporting Figure 3).

### *In Vitro* Binding Studies

Cellular uptake of the fluorescent conjugates was analyzed by flow cytometry according to published procedures.^21^ Briefly, 500 000 cells per cell line were incubated (triplicates) in 96-well plates (Greiner Bio-One) with 100 nM of MMC(IR800)-TOC or MMC(FNIR-Tag)-TOC for 1 h at 37 °C in the presence or absence of 100-fold excess octreotide, a potent SSTR2 agonist. After washing, cells were fixed in 4% formaldehyde and resuspended in 200 μL PBS. Untreated cells underwent the same procedure to determine background autofluorescence. Flow cytometry was performed with a NIRF-equipped BD FACSAria II, and median fluorescence intensity values were determined using the FlowJo software (BD).

For radioactive uptake studies, 200 000 cells (triplicates) were seeded in 96-well plates and incubated with 10 nM of ^67^Ga-DOTA-TOC, ^67^Ga-MMC(IR800)-TOC, or ^67^Ga-MMC(FNIR-Tag)-TOC in the presence (100×) or absence of octreotide for 1 h at 37 °C. After washing to remove unbound radioligand, cell-associated radioactivity was quantified using a Cobra II auto-γ counter (Packard), and the % of total radioactivity added was calculated from a known aliquot.

### Confocal Microscopy

Binding and internalization of the fluorescent conjugates were examined by confocal microscopy as previously described.^21^ Briefly, HCT116-SSTR2 cells were seeded in 8-well culture slides (Falcon) at a density of 100 000 cells/well. Following overnight attachment, cells were incubated with 5 μM MMC(IR800)-TOC or MMC(FNIR-Tag)-TOC in the presence or absence of 10-fold excess octreotide for 1 h at 37 °C. Cells were then washed with PBS, fixed in 4% formaldehyde, and mounted with Vectashield containing DAPI (Vector Laboratories). Fluorescence was detected using appropriate filter settings on a confocal microscope (Olympus FV3000) with excitation at 405 and 730 nm for the DAPI and NIRF signals, respectively.

### Tumor Imaging and Pharmacokinetic Analysis (Feasibility Study)

Mice with bilateral HCT116-SSTR2/WT xenografts (*n* = 5/group) were injected with 2 nmol (5.7 μg) of MMC(IR800)-TOC or MMC(FNIR-Tag)-TOC. *In vivo* imaging was performed with the In Vivo Xtreme (Bruker) preclinical imaging system at 1 and 3 h post-injection (p.i.). After euthanasia, tumors and tissues of interest were harvested for *ex vivo* imaging (3 h). Acquisition parameters for both *in vivo* and *ex vivo* imaging remained constant throughout the study: Excitation/Emission (Ex/Em) = 760 and 830 nm, respectively, exposure time = 10 s, binning = 4×, f-stop = 1.1, field-of-view = 1.9 cm. At the conclusion of macroscopic imaging, selected tissues were fixated and sectioned for immunohistopathological and mesoscopic imaging.

After euthanasia, one mouse was randomly selected from each group for cryo-fluorescence tomography (CFT) using the Xerra (Emit Imaging).^22^ Briefly, the mice were frozen in a cooling bath consisting of a hexanes/dry ice freezing mixture and embedded in OCT blocks. After mounting the block on the stage, the camera auto-adjusted to the best focus to capture white light and fluorescence images. The block was then sliced at 50 μm increments, and the automated process was repeated until the entire mouse was sectioned. Acquisition parameters were Ex/Em = 730 and 794 nm, respectively, and auto exposure was +3×.

### Dose and Time Point Optimization in NCI-H69 Xenografted Mice

NCI-H69 xenografts were injected with 2, 5, and 10 nmol of ^67^Ga-MMC(FNIR-Tag)-TOC (dual-labeled) and underwent *in vivo* (1 and 3 h) and *ex vivo* (3 h) NIRF imaging as detailed above (*n* = 4/group). Resected tissues were weighed, and γ counting was performed to quantitatively measure drug biodistribution as % of the injected activity per gram of tissue (%IA/g). The total injected radioactivity per mouse was determined from an aliquot of injected solutions. After analyzing results from the dose escalation study, 5 nmol was selected as the optimal dose, and the imaging/biodistribution study was repeated at 24 h p.i (*n* = 5). At the conclusion of γ counting, selected tissues were processed for immunohistopathological and mesoscopic imaging.

### *In Vivo* and *Ex Vivo* NIRF Imaging in a PDX Animal Model

Mice with bilateral NEC913/NEC1452 xenografts (*n* = 3) were injected with 5 nmol of MMC(FNIR-Tag)-TOC. *In vivo* and *ex vivo* NIRF imaging was conducted 24 h p.i. using the benchtop IVIS Lumina S5 small animal imaging system (PerkinElmer). Acquisition parameters were Ex/Em = 740 and 790 nm, respectively, small binning, subject height = 1.5 cm, F/Stop = 2, and exposure imaging times of 2 s for *in vivo* and 0.1 s for *ex vivo*. At the conclusion of macroscopic imaging, selected tissues were fixated and sectioned for immunohistopathological and mesoscopic imaging.

### Simulation of Proposed Clinical Workflow

Mice bearing orthotopically implanted BON–SSTR2 tumors (*n* = 5) were injected with 7.4 MBq (200 μCi; 0.5 nmol) of ^68^Ga-DOTA-TOC and underwent PET/computed tomography (CT) imaging (Albira small animal PET/CT scanner, Bruker) 1 h after injection. At 48 h after PET/CT imaging, mice were injected with 5 nmol of MMC(FNIR-Tag)-TOC, followed by *in vivo* NIRF imaging at 3 h p.i. as detailed above. After euthanasia, tumors were harvested under white light, and the wound bed was re-imaged with NIRF. After a more thorough visual inspection of the wound bed under white light, suspicious lesions were identified and harvested with the intact organ for *ex vivo* imaging along with other tissues of interest. Tumors, suspicious lesions, and relevant non-tumor tissues were processed for immunohistopathological and mesoscopic imaging.

### Image Analysis

Image analysis from NIRF *ex vivo* and CFT experiments were measured with the molecular Imaging (Bruker) and VivoQuant (Invicro) software, respectively. Tumor-to-background ratios (TBRs) were measured with respect to selected tissues of interest (i.e., NET-associated organs). TBR ratio was calculated using the formula , where *S*_t_ and *S*_b_ stand for fluorescent signal in tumor and background tissue, respectively.

For CFT two-dimensional (2D) and three-dimensional (3D) reconstruction, 2D images were obtained from individual slices, and an image stack was generated to produce 3D data sets. For PET/CT imaging, region-of-interest analysis was done with Integrated PMOD software (PMOD technologies) to standardize uptake values and determine TBRs.

### Histopathology and Mesoscopic NIRF Imaging

Tissues of interest (e.g., tumors, suspicious lesions, NET-associated organs) were embedded in paraffin or OCT to prepare formalin-fixed, paraffin-embedded (FFPE), or frozen sections, respectively. Blocks were then serially sectioned at 5 μm thickness, and one section per block was stained with standard hematoxylin and eosin (H&E). Immunohistochemistry (IHC) staining was performed on adjacent sections as we previously described.^17,21^ Briefly, after peroxidase inactivation, sections were incubated with anti-SSTR2 rabbit monoclonal antibody (ab134152, Abcam) overnight at 4 °C. After PBS washing, a secondary antibody (biotinylated goat anti-rabbit-polyvalent IgG) was applied for 10 min at room temperature. For visualization, a DAB detection kit (ab64261, Abcam) was used according to the manufacturer’s instructions. Sections were then counterstained with Mayer’s hematoxylin (Fisher Healthcare), dehydrated through two changes of alcohol, cleared in xylene, and cover-slipped with Cytoseal 60 mounting medium (Thermo Scientific). For FFPE sections, the slides were deparaffinized before H&E and IHC staining, and antigen retrieval was performed prior to IHC staining. For mesoscopic imaging, an adjacent section from each tissue was scanned on an Odyssey (LI-COR) at 800 nm with the highest resolution (21 μm).

### *Ex Vivo* Staining of Human Biospecimens with MMC(FNIR-Tag)-TOC

We obtained surgical biospecimens of pNETs (*n* = 5) and normal pancreas (*n* = 3) from the Institutional Tissue Bank at MD Anderson, whereas samples of lymph nodes (*n* = 2) and metastatic deposits from the liver (*n* = 2) were obtained from the Biospecimens Core at the University of Iowa. The use of surgical tissues was approved by the respective Institutional Review Boards of both institutes. Depending on availability, fresh or banked surgical biospecimens were used to prepare frozen sections. H&E and IHC staining were performed on consecutive sections as described above, and fluorescent staining followed published methods.^17,21^ In brief, frozen sections were thawed at room temperature and wetted with PBS. A 2 μM solution of MMC(FNIR-Tag)-TOC was then applied to the slides for 1 h at 37 °C. After incubation, the slides were washed with PBS, fixed in 4% paraformaldehyde, mounted with an antifade mounting medium (Vectashield, Vector Laboratories), and imaged as detailed above.

### Statistical Analysis

Statistical analysis was performed in GraphPad Prism 8.1.0. Group comparisons (*n* > 2) were performed with one- or two-way ANOVA along with multiple comparisons correction (Holm–Sidak). Family-wise significance and confidence levels were set to *P* < 0.05. All data are presented as mean ± SD. All *in vitro* experiments were performed with at least three technical and biological replicates.

## Results

### MMC-TOC Conjugates Have Similar Spectral Properties but Different Physicochemical Properties

MMC-TOC was produced according to prior methods and conjugated to DBCO-derivatives of IR800 and FNIR-Tag (Supporting Figure 1) using copper-free strain-promoted alkyne-azide cycloaddition (Supporting Figures 2 and 3).^16^ Following HPLC purification, spectral analysis revealed that both conjugates have excitation and emission peaks in the NIR region, with MMC(FNIR-Tag)-TOC having slightly blue-shifted spectra (λ_max_^em^ = 788 nm) compared to MMC(IR800)-TOC (λ_max_^em^ = 795 nm) due to differing C4′ substitution (Supporting Figure 4A). Both conjugates had the same fluorescence quantum yield (Φ_F_) of 0.13 in PBS. Although both probes have similar optical properties, they have markedly different physicochemical properties. Upon conjugation of the fluorophores to the MMC, the net overall charge differs for MMC(IR800)-TOC (-5) and MMC(FNIR-Tag)-TOC (-2). cLog P values highlight that MMC(FNIR-Tag)-TOC (−6.68) is much more hydrophilic than MMC(IR800)-TOC (0.0938), suggesting that MMC(FNIR-Tag)-TOC will have improved *in vivo* performance. Spectral and physicochemical properties are summarized in Supporting Figure 4B.

### MMC(FNIR-Tag)-TOC Maintains SSTR2-Targeted Binding Properties with Lower Nonspecific Uptake *In Vitro*

To evaluate the binding properties of MMC(FNIR-Tag)-TOC, we performed *in vitro* experiments and used the IR800 counterpart as a control. We selected HCT116-WT (no SSTR2), HCT116-SSTR2, BON–SSTR2, and NCI-H69 cells based on their varying SSTR2 expression (HCT116-SSTR2 ≫ BON–SSTR2 > NCI-H69) (Supporting Figure 5) and established use for characterizing novel SSTR2-targeted agents.^17,23−26^ Flow cytometry results showed comparable uptake between agents in HCT116-SSTR2 cells that was reduced when coincubated with octreotide and in the absence of SSTR2 (HCT116-WT cells) (Figure 1A). Notably, we found a >2.5-fold increase in specific binding ratio with the FNIR-Tag conjugate (12.2 and 33.6 for IR800 and FNIR-Tag, respectively), indicating lower nonspecific interactions with the charge-balanced dye (Supporting Figure 6A). Although the IR800 analogue had higher uptake in BON–SSTR2 and NCI-H69 cell lines, it also had higher nonspecific uptake, as shown by blocking studies, which yielded comparable specific binding ratios (1.3–2.8). We then applied the radioactive utility of the MMC to compare ^67^Ga-labeled FNIR-Tag and IR800 conjugates to the gold standard, ^67^Ga-DOTA-TOC. Both radiolabeled conjugates had uptake (range on average, 7.7–8.3%) comparable to the positive control, ^67^Ga-DOTA-TOC (8.7 ± 1.8%) in HCT116-SSTR2 cells (Figure 1B). Interestingly, the binding characteristics of ^67^Ga-MMC(FNIR-Tag)-TOC were more representative of ^67^Ga-DOTA-TOC across cell lines and translated into specific binding ratios of 9.2 (HCT116-SSTR2), 5.9 (BON–SSTR2), and 1.7 (NCI-H69), which compare favorably to the clinical agent (10, 20, and 4.4, respectively) and are higher than those of ^67^Ga-MMC(IR800)-TOC (4.5, 1.7, and 1.0, respectively) (Supporting Figure 6B). To confirm that FNIR-Tag conjugation does not impair the agonist properties of TOC (*e.g.*, receptor internalization), we performed confocal microscopy in HCT116-SSTR2 cells and observed SSTR2-mediated internalization that was similar to MMC(IR800)-TOC (Figure 1C). Collectively, the *in vitro* data shows that replacing IR800 with FNIR-Tag produces a bioactive conjugate with high selectivity for SSTR2-expressing cells.

### FNIR-Tag Provides Superior *In Vivo* Performance and Increases SSTR2-Mediated Tumor Contrast at Early Time Points

**Figure 2:**
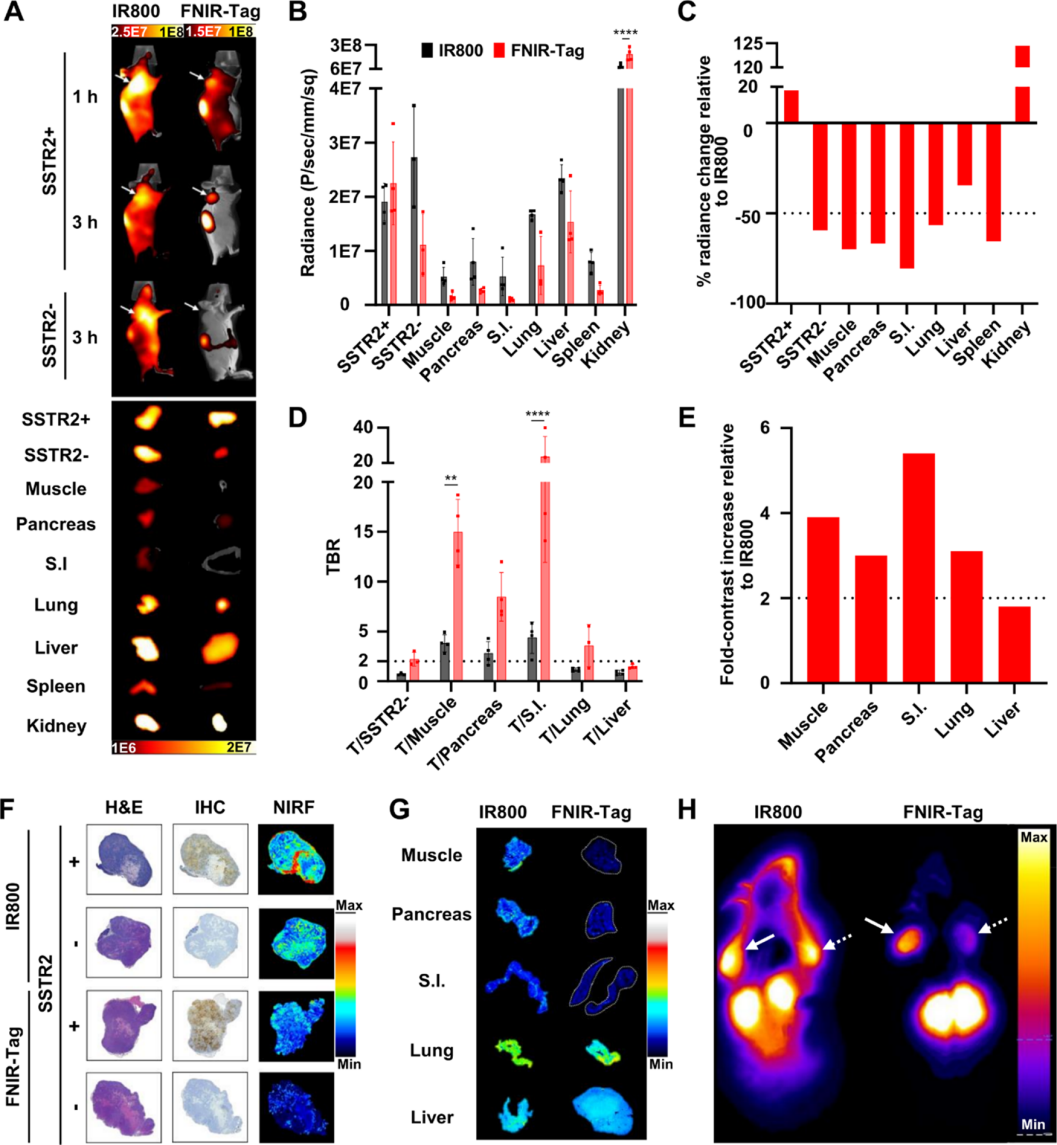
*In vivo* and multiscale comparison of MMC(FNIR-Tag)-TOC and MMC(IR800)-TOC. (A) *In vivo* (1 and 3 h) and *ex vivo* (3 h) NIRF imaging (*in vivo* Xtreme, Bruker) in the HCT116-SSTR2/WT (SSTR2±) dual implant animal model after injection of 2 nmol MMC(IR800)-TOC or MMC(FNIR-Tag)-TOC; arrows indicate tumors; imaging scale, photons/sec/mm^2^; S.I., small intestine. *Ex vivo* image analysis of selected tissues as determined by (B) fluorescence output and (D) TBR, or by changes in (C) % signal and (E) fold-contrast in MMC(FNIR-Tag)-TOC cohorts relative to the IR800 conjugate. Results are presented as mean ± s.d. (*n* = 4/group) except for (C) and (E), which are shown as data on average. ^****^P ≤ 0.0001. ^**^P ≤ 0.01. (F) Immunohistopathology and mesoscopic NIRF imaging of SSTR2± tumors or (G) normal tissues relevant to NET surgery (NIRF imaging only). (H) Axial slice of CFT (Xerra, Emit) comparing conjugates while maintaining anatomical context. Solid and dashed arrows indicate SSTR2+ and SSTR2– tumors, respectively. CFTs for both agents are scaled equally.

The objective of our initial *in vivo* study was to demonstrate the feasibility of SSTR2 targeting and assess dye effects on imaging properties. Accordingly, we selected the dually implanted HCT116-SSTR2/WT xenograft model and a dose of 2 nmol per mouse, which we previously used to evaluate our first-generation agent.^17^ We injected the fluorescent conjugates in mice and performed *in vivo* NIRF imaging at 1 and 3 h p.i., followed by *ex vivo* imaging of resected tissues. Consistent with our published results,^17^ clear tumor delineation was not possible with MMC(IR800)-TOC at either time point due to high background fluorescence (Figure 2A, Supporting Figures 7 and 8). Conversely, the reduction in background signal shown with other zwitterionic dye-conjugates^3^ was evident with MMC(FNIR-Tag)-TOC and provided excellent SSTR2-mediated tumor localization at 3 h. *Ex vivo* imaging showed comparable fluorescence in SSTR2+ tumors with both conjugates, but in the absence of SSTR2, only the FNIR-Tag conjugate produced the expected decrease in tumor signal, an observation that extended to all normal tissues except the kidney (Figure 2A). Semiquantitative analysis of *ex vivo* imaging supported qualitative observations (Figure 2B, Supporting Figure 9). We measured, on average, a slight increase in tumor uptake with MMC(FNIR-Tag)-TOC and a >60% signal reduction in tumors lacking SSTR2 and in most nontarget tissues, including those associated with NET surgery (e.g., pancreas, small intestine) (Figure 2C). This effect translated into TBRs >8.0 in the pancreas and small intestine, representing a >3.5-fold increase relative to our first-generation agent (Figure 2D–E).

Fluorescence imaging at the macroscale is subjected to the diffuse nature of photons and may obscure the true specificity of FGS agents that target a tumor biomarker (*e.g.*, a receptor).^27^ Thus, we examined the correlation between fluorescence and SSTR2 distribution using mesoscopic NIRF imaging and immunohistopathology. In tumors, we found that only MMC(FNIR-Tag)-TOC had a fluorescence localization pattern that was consistent with IHC staining (Figure 2F). This finding suggests a low degree of nonspecific interactions in the tumor milieu and, importantly, minimal contribution of the enhanced permeability and retention (EPR) effect on agent accumulation. NIRF imaging of normal tissue sections also showed lower background with the second-generation conjugate (Figure 2G). Next, we performed whole-body CFT to map out the distribution and signal intensity of both conjugates while maintaining anatomical context. As shown in a representative axial slice, the lower nonspecific binding of MMC(FNIR-Tag)-TOC relative to the IR800 analogue produced a markedly lower background fluorescence along with a strong tumor signal only in the presence of SSTR2 (Figure 2H). Analysis of tumor regions revealed 2-fold higher signal in SSTR2+ tumors for mice receiving MMC(FNIR-Tag)-TOC, whereas the cohort injected with the IR800 conjugate had identical fluorescence intensities in both tumors (Supporting Figure 10). The cumulative effects of dye optimization were illustrated using 2D and 3D reconstructions that clearly support the use of the second-generation agent for high-contrast *in vivo* imaging (Multimedia Files 1 and 2). Overall, these findings demonstrate that MMC(FNIR-Tag)-TOC has higher tumor specificity and more favorable pharmacokinetics than its IR800 counterpart, which could translate into increased tumor contrast along lower false-positive rates in a translational setting, thereby enhancing surgical accuracy.

### SSTR2-Mediated Delivery of FNIR-Tag Provides High and Similar Tumor Contrast Independent of Dose and Time

**Figure 3:**
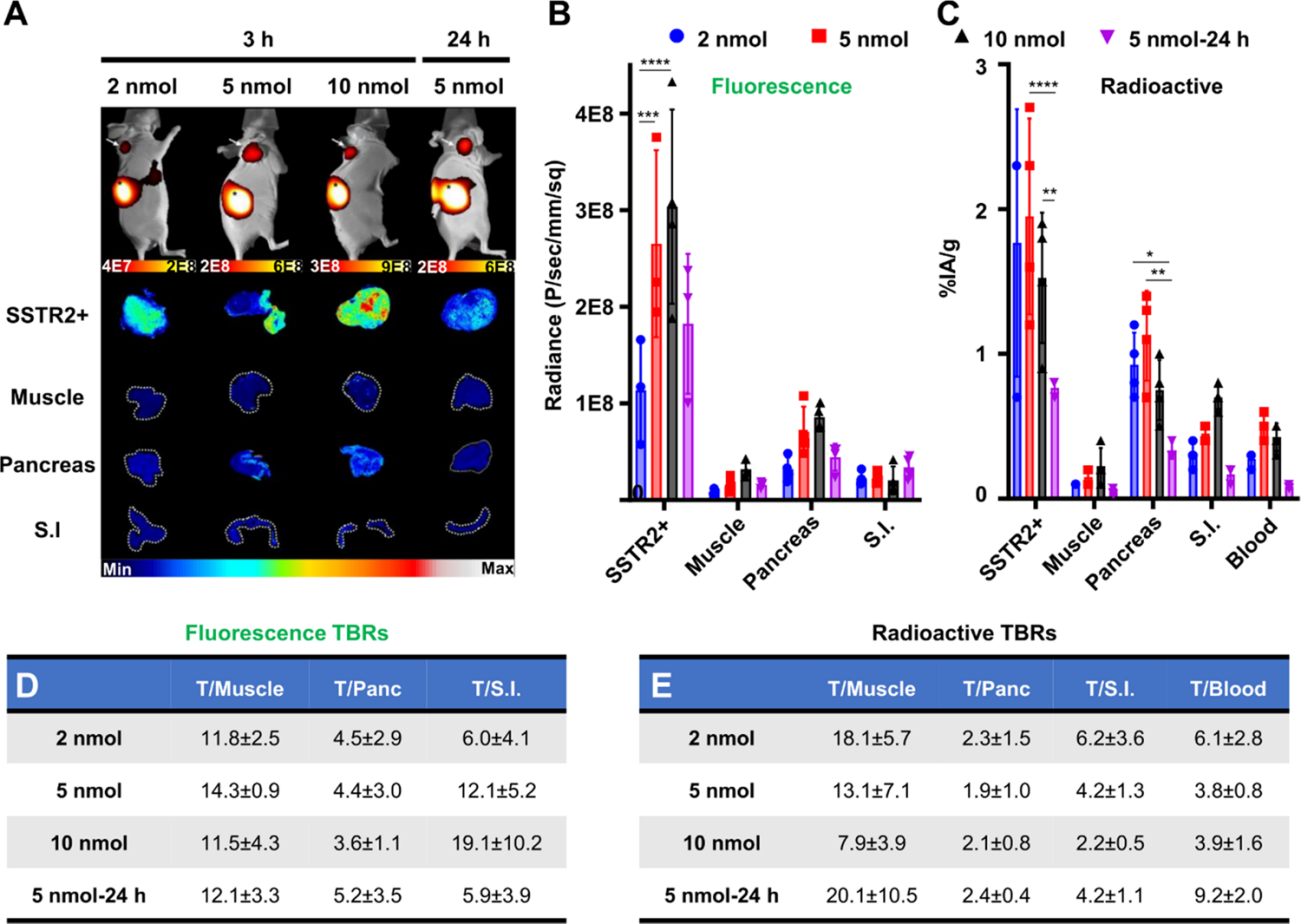
Dose and time effect on ^67^Ga-MMC(FNIR-Tag)-TOC pharmacokinetics in NCI-H69 xenografts. (A) *In vivo* and mesoscopic NIRF imaging (3 or 24 h; *in vivo* Xtreme, Bruker) after injection of 2, 5, or 10 nmol of radiolabeled conjugate. Arrows indicate tumors; imaging scale, photons/sec/mm^2^; SSTR2+, xenograft tumors; S.I., small intestine. Analysis of conjugate biodistribution by (B) fluorescence (semiquantitative) and (C) radioactive (quantitative) uptake in NET-related organs (pancreas and S.I.) and nontarget tissues (muscle and blood). Results are presented as mean ± s.d. (*n* = 4/dose and time). ^****^P ≤ 0.0001, ^***^P ≤ 0.001, ^**^P ≤ 0.01, and *P < 0.05. TBRs measured by the (D) fluorescence and (E) radioactive signal.

Dose and time play an important role in determining tumor contrast and can be optimized to strengthen the predictive value of an FGS agent.^28−30^ Since fluorescence emits low-energy photons that limit the measurement of absolute drug concentration,^31^ we radiolabeled MMC(FNIR-Tag)-TOC with the γ-emitting radionuclide ^67^Ga to overcome attenuation and scattering phenomena.^32,33^ We injected increasing doses (2, 5, and 10 nmol) of ^67^Ga-MMC(FNIR-Tag)-TOC into nude mice with NCI-H69 xenografts, which endogenously express SSTR2, and imaged at 3 and 24 h p.i. Figure 3A qualitatively illustrates that tumor uptake increased as a function of dose while decreasing with time both at the macro- and mesoscopic scales; importantly, tumor signal was the highest among nonclearance organs regardless of dose or imaging time. NIRF imaging of complete cohorts is shown Supporting Figure 11. The qualitative correlation between fluorescence and SSTR2 distribution (IHC) as a function of dose and time is shown in Supporting Figure 12. Semiquantitative analysis of fluorescence readouts (Figure 3B) supported imaging findings, and quantitative analysis of radioactive readouts cross-validated the general fluorescence profile while standardizing drug uptake as %IA/g of tissue (Figure 3C). Overall, both detection modalities showed that agent uptake in tumor and non-tumor tissues varied slightly as a function of dose (Supporting Tables 1 and 2), suggesting saturable tumor binding (1.5–2.0%IA/g) and efficient clearance (<0.25 and <0.50%IA/g in muscle and blood at 3 h, respectively). On average, fluorescent TBRs were >3.5 and >5.5 in the pancreas and small intestine, respectively, with no differences (*P* > 0.05) associated with dose escalation (Figure 3D). Thus, we selected 5 nmol as the dose for delayed imaging based on the combination of high tumor fluorescence and low background signal. Despite increased agent washout from normal organs at 24 h, a slight decrease in tumor signal produced TBRs that were similar to 3 h imaging (Figure 3D). Radioactive TBRs were consistent with fluorescent-based results (Figure 3E). From these experiments, we identified 5 nmol and 3 h as the preferred dose and imaging time point for MMC(FNIR-Tag)-TOC and applied those conditions to subsequent *in vivo* studies.

### MMC(FNIR-Tag)-TOC Exhibits SSTR2-Mediated Binding in Human Tumor Tissues

**Figure 4:**
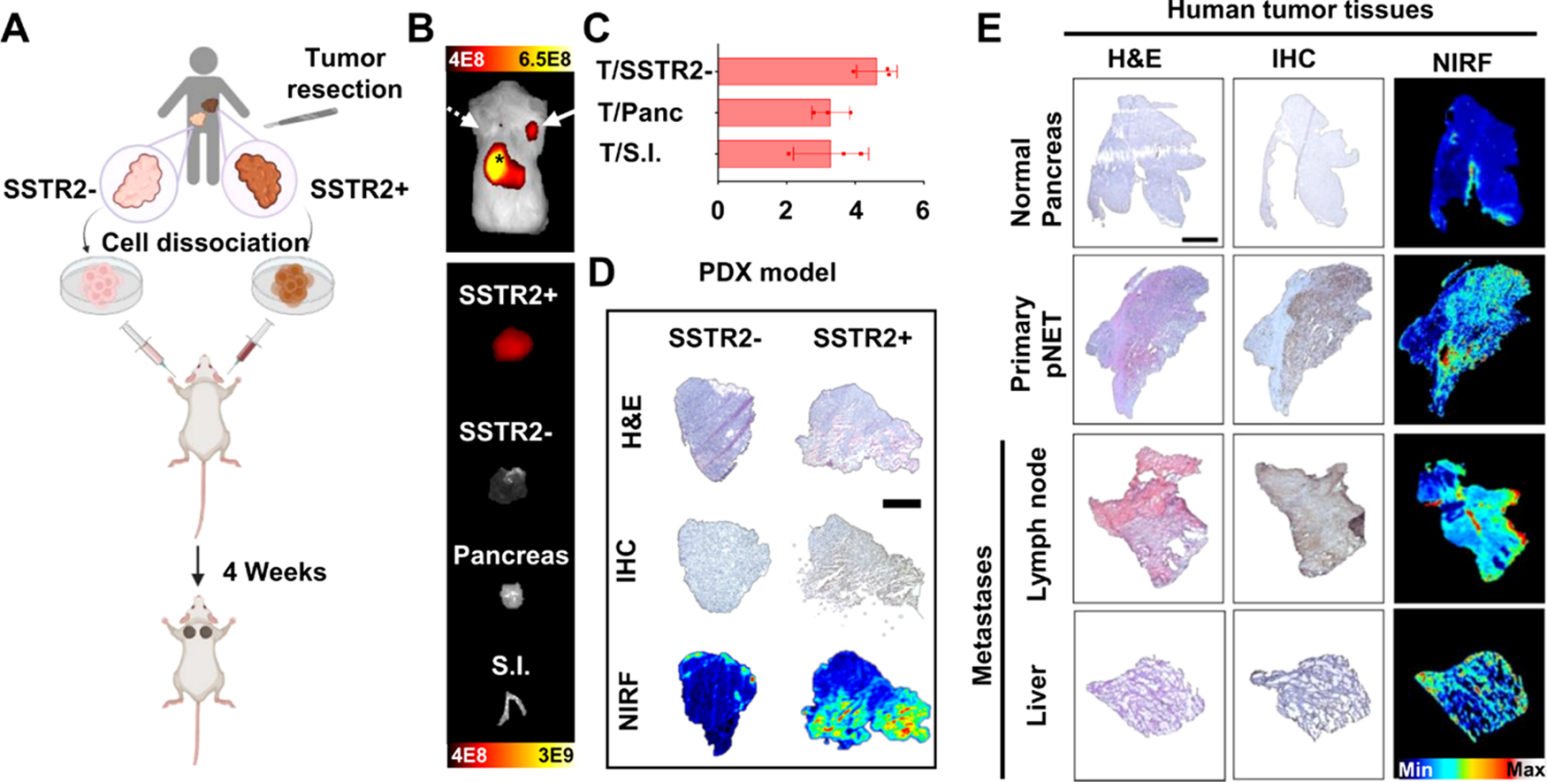
MMC(FNIR-Tag)-TOC binding in a PDX model and human biospecimens. (A) Schematic showing the development of a novel neuroendocrine carcinoma PDX model, NEC931/NEC1452 (SSTR2±). Representative (B, top) *in vivo* and (B, bottom) *ex vivo* NIRF images (IVIS, PerkinElmer) after injection of 5 nmol MMC(FNIR-Tag)-TOC at 24 h p.i. Solid and dashed arrows indicate SSTR2+ and SSTR2– tumors, respectively. Imaging scale, [Photons/sec/cm^2^]/[μW/cm^2^]. S.I., small intestine. Panc., pancreas. (C) Tumor-to-tissue ratios in SSTR2- tumors and selected NET-relevant tissues (pancreas, small intestine). Results are presented as mean ± s.d. (*n* = 3/group). (D) Confirmation of selective accumulation of the agent in SSTR2+ PDX tumors by NIRF imaging and immunohistopathology assessment of frozen sections. (E) *Ex vivo* staining of human primary and metastatic NET sections with the fluorescence probe. IHC and H&E staining were performed to provide SSTR2 distribution and morphologic references, respectively. Scale is 200 μm.

To evaluate the translational potential of MMC(FNIR-Tag)-TOC, we first used the novel neuroendocrine carcinoma (NEC) patient-derived xenograft (PDX) models, NEC913 (SSTR2+) and NEC1452 (SSTR2−), that more accurately recapitulate human disease^34^ (Figure 4A). We injected dually implanted PDX mice with 5 nmol of MMC(FNIR-Tag)-TOC and performed *in vivo* and *ex vivo* NIRF imaging at 24 h p.i. As shown in Figure 4B, agent accumulation was only observed in SSTR2+ tumors, with minimum to no signal in tumors lacking the receptor or in normal tissues. Image analysis revealed a >3-fold higher uptake in SSTR2+ tumors compared with SSTR2- tumors, pancreas, and small intestine (Figure 4C). Mesoscopic NIRF imaging was in accordance with *in vivo* and *ex vivo* results, and signal distribution corresponded to IHC-positive areas, demonstrating high specificity of the agent for SSTR2-expressing tissues (Figure 4D). Given the confounding effects of tumor heterogeneity in the clinical setting, we examined the robustness of our SSTR2-targeted FGS strategy using frozen sections from freshly resected pNETs (with adjacent normal tissues), metastatic lesions (liver), and lymph nodes. After confirming tumor histology with H&E staining and SSTR2 expression by IHC, we incubated adjacent tissue sections with MMC(FNIR-Tag)-TOC and obtained fluorescence readouts using the Odyssey NIR imager. Slide scanning showed excellent colocalization of agent binding with IHC staining and demonstrated the ability of MMC(FNIR-Tag)-TOC to target (i) primary pNETs from patients, (ii) metastases that express SSTR2, and (iii) involved lymph nodes (Figure 4E). Overall, these findings demonstrated the excellent SSTR2-targeting properties of MMC(FNIR-Tag)-TOC in tissues most often affected by GEP-NETs and showed the translational potential of this probe in facilitating FGS.

### MMC(FNIR-Tag)-TOC Translates Preoperative Imaging Findings into the Operating Room and Reveals Unknown Metastatic Deposits

**Figure 5:**
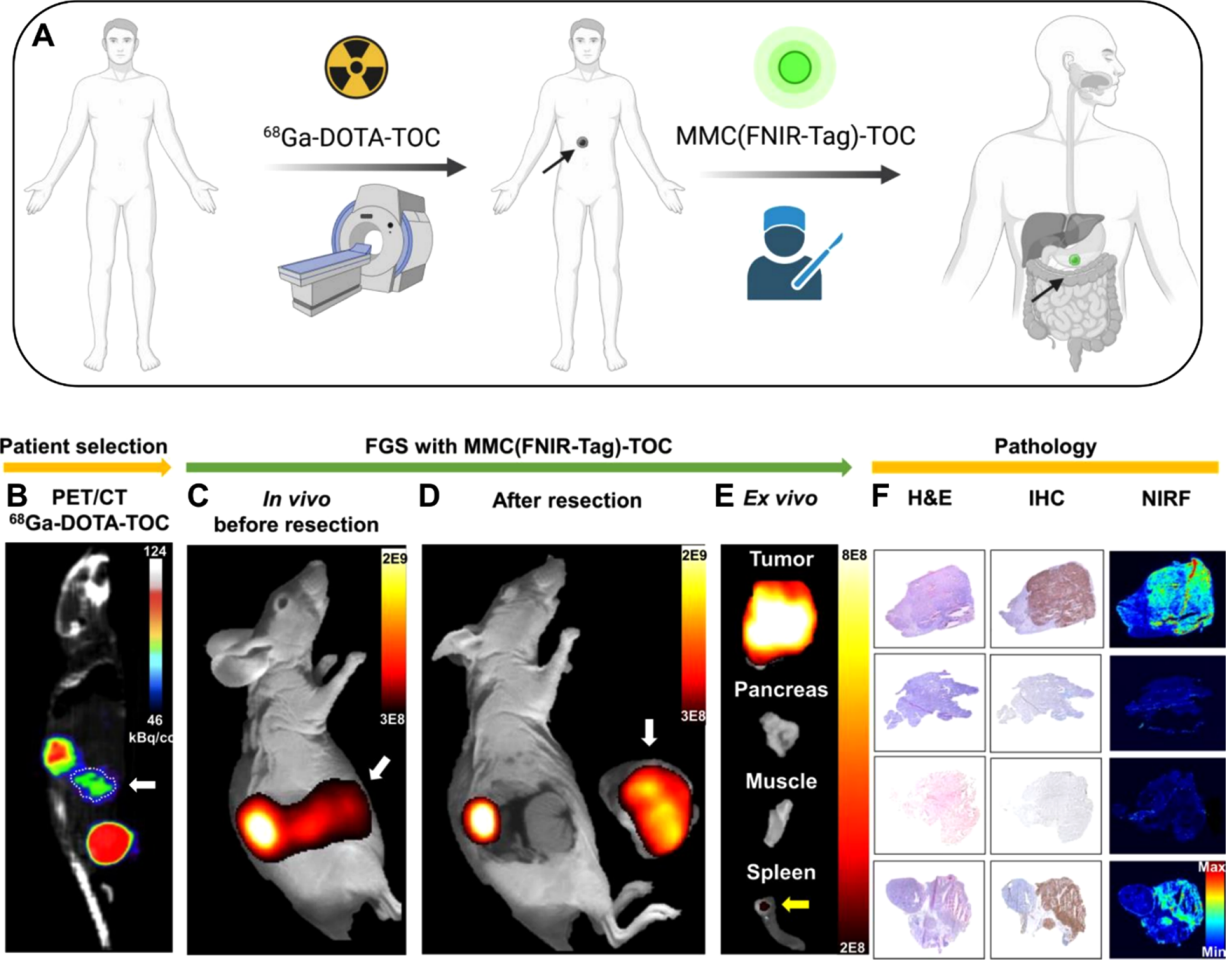
Translating the utility of targeted preoperative NET imaging into the operating room—a simulation. (A) Schematic of a proposed PET-based clinical workflow to determine patient eligibility for SSTR2-targeted FGS. (B) Representative “patient” selection with the PET/CT gold standard, ^68^Ga-DOTA-TOC, in a BON–SSTR2 orthotopic pancreatic animal model showed unexpected tracer uptake in the gut region. (C) *In vivo* NIRF imaging with MMC(FNIR-Tag)-TOC is in accordance with PET/CT and (D) demonstrated complete tumor resection *via* the absence of fluorescence in the wound bed. (E) *Ex vivo* imaging shows fluorescence only in tumor and spleen metastasis (yellow arrow), with pancreas and muscle signals at background levels. (F) Agent specificity for SSTR2 and fluorescence distribution in tumors (mesoscopic NIRF imaging) are validated with IHC and the surgical gold standard (H&E), respectively. White arrows indicate tumor.

To further examine the translational value, we generated an orthotopic tumor model by injecting BON–SSTR2 cells into the pancreas of athymic nude mice.^18^ Eight weeks after implantation, we implemented a proposed clinical workflow modeled after the theranostic paradigm that uses ^68^Ga-DOTA-TOC (or ^68^Ga-DOTA-TATE) as a patient selection tool for patients who may benefit from peptide-receptor radionuclide therapy (Figure 5A).^35,36^ We performed PET/CT imaging with ^68^Ga-DOTA-TOC to represent preoperative identification of SSTR2+ disease that would potentially benefit from intraoperative imaging with MMC(FNIR-Tag)-TOC. Abnormal uptake was observed in the abdomen of 4 out of 5 mice (representative mouse shown in Figure 5B) and suggested tumor development in the pancreas. Two days later, we injected mice with 5 nmol of MMC(FNIR-Tag)-TOC, followed by NIRF imaging and surgical resection at 3 h p.i. *In vivo* NIRF imaging showed strong fluorescence in the gut in 4 of 5 mice that was in accordance with PET/CT findings (Figure 5C). We then used visual inspection under white light to locate and resect the tumor and confirmed complete resection by NIRF imaging of the tumor bed (Figure 5D). *Ex vivo* imaging (Figure 5E) yielded a tumor-to-pancreas ratio of 17.7 ± 9.3, suggesting excellent potential for visual contrast in an intraoperative setting. In addition to the primary tumor site, we also detected unexpected fluorescence in the spleen that was suspected to be metastatic disease based on prior reports with this animal model.^37,38^ Analysis of tumor and suspicious lesions confirmed cancer status (H&E) and SSTR2-positive regions (IHC) that correlated with fluorescence (Figure 5F). These findings demonstrate high detection sensitivity of both primary and metastatic lesions using MMC(FNIR-Tag)-TOC and suggest excellent potential for detecting subclinical metastatic deposits and post-resection residual disease that would have otherwise been missed.

## Discussion

The somatostatin analogue/SSTR2 ligand-receptor axis is the central component of theranostic approaches in GEP-NETs. Conceivably, adapting SSTR2-targeted peptides for FGS could broaden the theranostic utility by extending the visual information of preoperative PET imaging into the operating room. This would require similar *in vivo* performance (*i.e.*, targeting and pharmacokinetic properties) between the intraoperative contrast agent and the clinically approved PET radiotracer, ^68^Ga-DOTA-TOC (or ^68^Ga-DOTA-TATE). However, converting a radiopeptide into a fluorescent counterpart is complicated by factors such as (i) a significant increase in molecular weight that can slow excretion, (ii) higher lipophilicity that shifts clearance from the kidneys, which are preferred, to the liver, and (iii) a poor charge-to-hydrophobicity distribution that is imparted by commonly used NIRF dyes.^39,40^ For our first-generation FGS agent (^68^Ga-MMC(IR800)-TOC), these collective effects resulted in longer blood residence time compared with ^68^Ga-DOTA-TOC^16^ and required delayed imaging (24–48 h p.i.) to obtain meaningful contrast.^17^ We also observed higher clearance *via* the reticuloendothelial system (liver and spleen), which has significant clinical implications since it could reduce detection sensitivity for metastases in these organs.^28^

While size and lipophilicity are intrinsic properties of cyanine dyes, multiple strategies have been developed to address the effects of surface charge. ZW800-1 is a zwitterionic cyanine designed to provide a balanced charge distribution with a net charge of 0 after conjugation.^2^ Head-to-head studies showed that ZW800-1 conjugates had lower serum binding and nonspecific tissue uptake compared with their anionic counterparts, resulting in higher TBRs.^3^ More recently, the charge-balanced, symmetrical, and PEGylated cyanine FNIR-Tag was developed to address the problem of aggregation-induced quenching that occurs with IR800-labeled antibodies.^7^ Antibodies labeled with FNIR-Tag had higher tumor uptake, reduced liver uptake, and enhanced brightness compared with IR800-labeled conjugates. Unlike IR800, FNIR-Tag reduces fluorescence signal in blood while minimizing interactions with serum proteins and cationic surfaces. To achieve the combination of high tumor binding and low background signal associated with radiolabeled somatostatin analogues, we developed the second-generation FGS agent MMC(FNIR-Tag)-TOC and demonstrated its superior performance in cells, animal models, and human biospecimens. The mechanism by which FNIR-Tag mitigates nonspecific interactions and enhances tumor contrast relative to IR800 remains to be investigated. Previous studies showed that C4′-*O*-alkyl cyanines (*i.e.*, FNIR-Tag-like dyes) are less reactive to cellular proteins (*e.g.*, C4′-thio adduct formation) than the C4′-*O*-aryl IR800.^41−43^ Here, we found that MMC(FNIR-Tag)-TOC retains tumor targeting, decreases background fluorescence in liver and spleen (30–60%), and increases >2-fold kidney signal compared to the IR800 analogue (Figure 2C). The strong and persistent kidney signal (Figure 3A) suggests a shift in clearance pathways and is consistent with PET readouts from patients injected with radiolabeled somatostatin analogues, where a combination of tubular reabsorption *via* the megalin/cubilin receptor complex and SSTR2-mediated reabsorption in the glomeruli and renal tubule cells occurs.^44−47^

Several low-molecular-weight FGS agents have advanced to clinical trials,^48−50^ and the folate receptor-targeted agent OTL-38^30^ recently gained regulatory approval for intraoperative identification of ovarian cancer. Given the similar need for intraoperative guidance during GEP-NET surgery, we systematically examined the translational utility of MMC(FNIR-Tag)-TOC in the present study. Most notably, we demonstrated that employing a charge-balanced dye restored the low background signal of the parent peptide in multiple tumor models and produced a >60% decrease in fluorescence signal in the pancreas and small intestine. The lower off-target signal also improved TBRs to the extent that it was possible to visualize tumors as early as 3 h p. i. These combined effects could conceivably increase the positive predictive value of SSTR2-targeted FGS and expand the imaging time window to give surgeons flexibility in administering MMC(FNIR-Tag)-TOC. While these findings suggest an improved likelihood for intraoperative tumor delineation with the second-generation conjugate, we acknowledge that results obtained with preclinical imaging systems are not indicative of utility with a clinical imaging device.^51^ However, we previously addressed this limitation by showing the feasibility of tumor imaging with our first-generation agent in combination with the da Vinci Firefly imaging system and a custom-built clinical prototype NIRF imaging system (OnLume Inc.) and anticipate similar drug-device compatibility with MMC(FNIR-Tag)-TOC.^17,28,52^ We also demonstrated the unique benefit of selecting DOTA-TOC as the foundation for the fluorescent analogue, as it allowed the use of standard-of-care PET imaging for “patient” selection in animal studies. This strategy is currently not possible with any other FGS agents since they do not have an FDA-approved nuclear imaging counterpart and may instead rely on biopsies or alternative imaging techniques to determine target expression.

In conclusion, our data shows that the engineered dye FNIR-Tag can produce an SSTR2-targeted FGS agent with superior tumor specificity. Clinical application of our agent may enable the detection and removal of multifocal small bowel NETs and nodal disease that may otherwise be missed with standard surgical techniques. SSTR2-targeted FGS also has the potential to increase surgical accuracy in pNETs by facilitating parenchyma-sparing R0 resections. This capability could reduce unnecessarily wide surgical margins that can cause pancreatic insufficiency in up to 40% of patients and impair quality of life.^53−55^ Furthermore, adding real-time tumor imaging to minimally invasive (robotic) surgery may compensate for its lack of tactile feedback. Given the existing use of nontargeted NIRF dyes with commercially available surgical robotic systems, similar integration of MMC(FNIR-Tag)-TOC is feasible and could facilitate quicker recovery after GEP-NET surgery.^56^ Finally, our FGS strategy could potentially extend beyond GEP-NETs by facilitating the conversion of other radiolabeled peptides and small molecules into tumor-targeted fluorescent analogues. Thus, new imaging capabilities would become available for a variety of tumor types that rely on surgery as their primary treatment modality.

## Supporting Information Available

Synthesis of FNIR-Tag-DBCO; chemical structure of MMC(FNIR-Tag)-TOC; (radio)HPLC chromatograms; spectral and physicochemical properties of fluorescent conjugates; supporting material related to *in vitro* and *in vivo* experiments; and radioactive and fluorescent biodistribution of ^67^Ga-MMC(FNIR-Tag)-TOC as a function of dose and time (PDF)
2D CFT reconstruction (Xerra, Emit) after injection of 2 nmol MMC(IR800)-TOC (left) and MMC(FNIR-Tag)-TOC (right) at 3 h p.i. in the HCT116-SSTR2/WT dual implant animal model; solid and dashed arrows indicate SSTR2+ and SSTR2– tumors, respectively; CFTs for both agents are scaled equally; the original video was compressed to meet publication standards and may have resulted in loss of quality (MP4, 16 Mb) (Multimedia File 1) (MP4)
3D CFT reconstruction (Xerra, Emit) after injection of 2 nmol MMC(IR800)-TOC (left) and MMC(FNIR-Tag)-TOC (right) at 3 h p.i. in the HCT116-SSTR2/WT dual implant animal model; SSTR2+ and SSTR2– tumors are implanted on the left and right shoulder of each mouse, respectively; CFTs for both agents are scaled equally; the original video was compressed to meet publication standards and may have resulted in loss of quality (MP4, 10 Mb) (Multimedia File 2) (MP4)
