# Supplementary material for: High-Contrast Detection of Somatostatin Receptor Subtype-2 for Fluorescence-Guided Surgery
Source: Mol Pharm. 2022 Sep 29;19(11):4241–53. doi: 10.1021/acs.molpharmaceut.2c00583 (PMC9830638; doi:10.1021/acs.molpharmaceut.2c00583)
Supplement: Supplementary file 1 — mp2c00583_si_001.pdf [file mp2c00583_si_001.pdf]

Supporting information

High-Contrast Detection of Somatostatin Receptor Subtype-2 for Fluorescence-Guided Surgery

Servando Hernandez Vargas[1§], Solmaz AghaAmiri[1§], Sukhen C. Ghosh[1§], Michael P. Luciano[2], Luis C. Borbon[3], Po Hien Ear[3], James R. Howe[3], Jennifer M. Bailey-Lundberg[4], Gregory D. Simonek[5], Daniel M. Halperin[6], Hop S. Tran Cao[7], Naruhiko Ikoma[7], Martin J. Schnermann[2], and Ali Azhdarinia[1*]

[1]The Brown Foundation Institute of Molecular Medicine, McGovern Medical School, The University of Texas Health Science Center at Houston, Houston, Texas 77054, USA.

[2]Chemical Biology Laboratory, Center for Cancer Research, National Cancer Institute, Frederick, MD, 21702, USA.

[3]Department of Surgery, University of Iowa Carver College of Medicine, Iowa City, Iowa, 52242, USA.

[4]Department of Anesthesiology, McGovern Medical School, The University of Texas Health Science Center at Houston, Texas 77030, USA.

[5]Center for Laboratory Animal Medicine and Care, McGovern Medical School, The University of Texas Health Science Center at Houston, Texas 77030, USA.

[6]Department of Gastrointestinal Medical Oncology, The University of Texas MD Anderson Cancer Center, 1515 Holcombe Blvd., Houston, TX, 77030, USA.

[7]Department of Surgical Oncology, The University of Texas MD Anderson Cancer Center, 1515 Holcombe Blvd., Houston, TX, 77030, USA.

[§]These authors contributed equally.

Running Title: High contrast detection of SSTR2 for FGS

Keywords: fluorescence-guided surgery, intraoperative imaging, cancer surgery, dual labeling, near-infrared fluorescence imaging, somatostatin receptor

The authors declare no potential conflicts of interest.

*To whom correspondence should be addressed:
Ali Azhdarinia, Ph.D.
The Brown Foundation Institute of Molecular Medicine
University of Texas Health Science Center at Houston
1881 East Road. 3SCR6.4680, Houston, TX 77054
Office: 713-500-3577
email: ali.azhdarinia@uth.tmc.edu

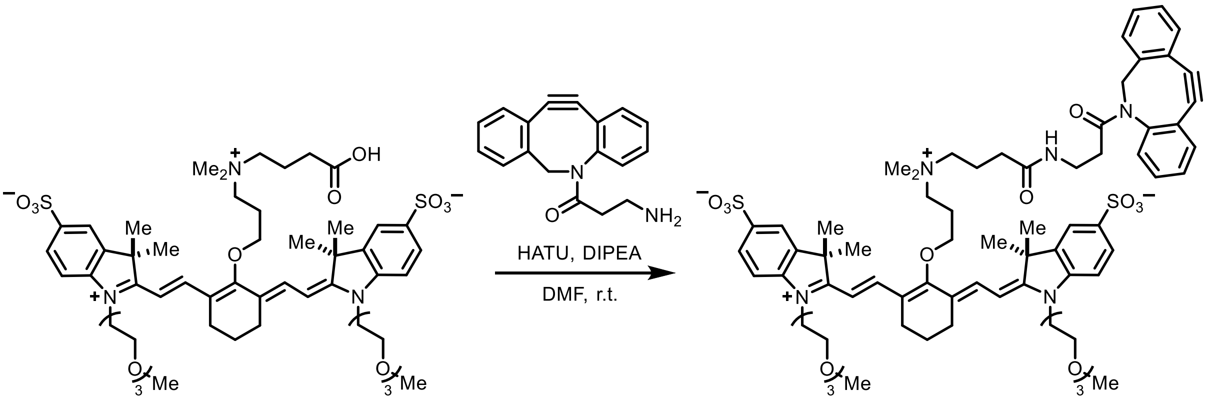

FNIR-Tag **FNIR-Tag-DBCO**

Supporting Figure 1. Synthesis of FNIR-Tag-DBCO.

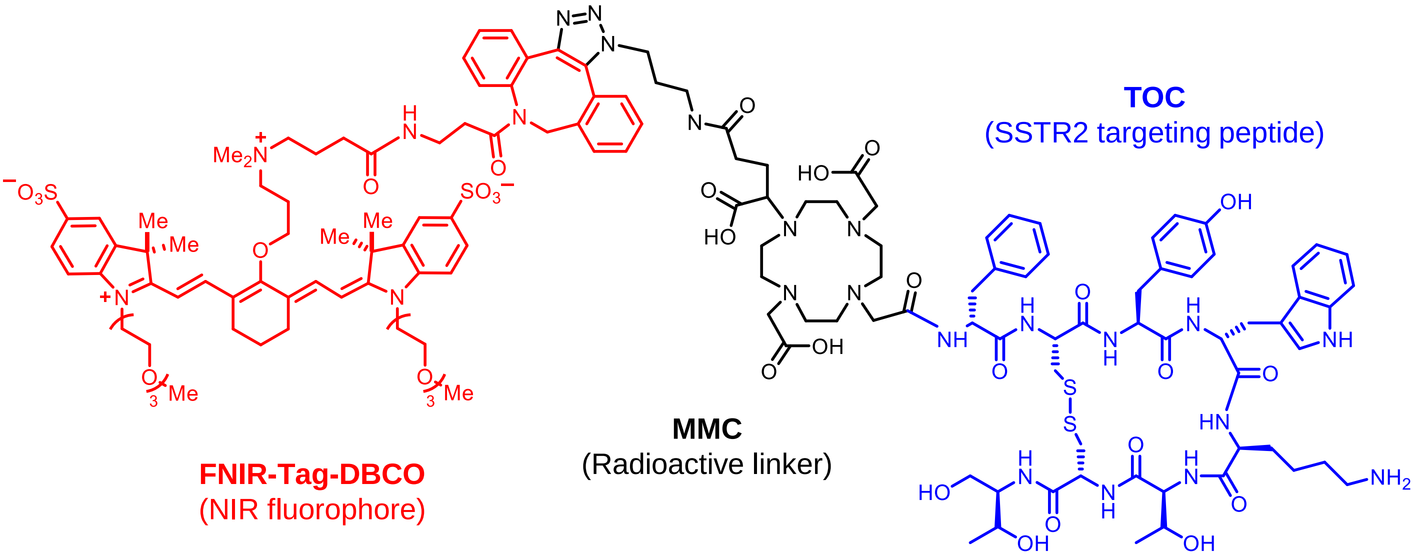

Supporting Figure 2. Chemical structure of MMC(FNIR-Tag)-TOC.

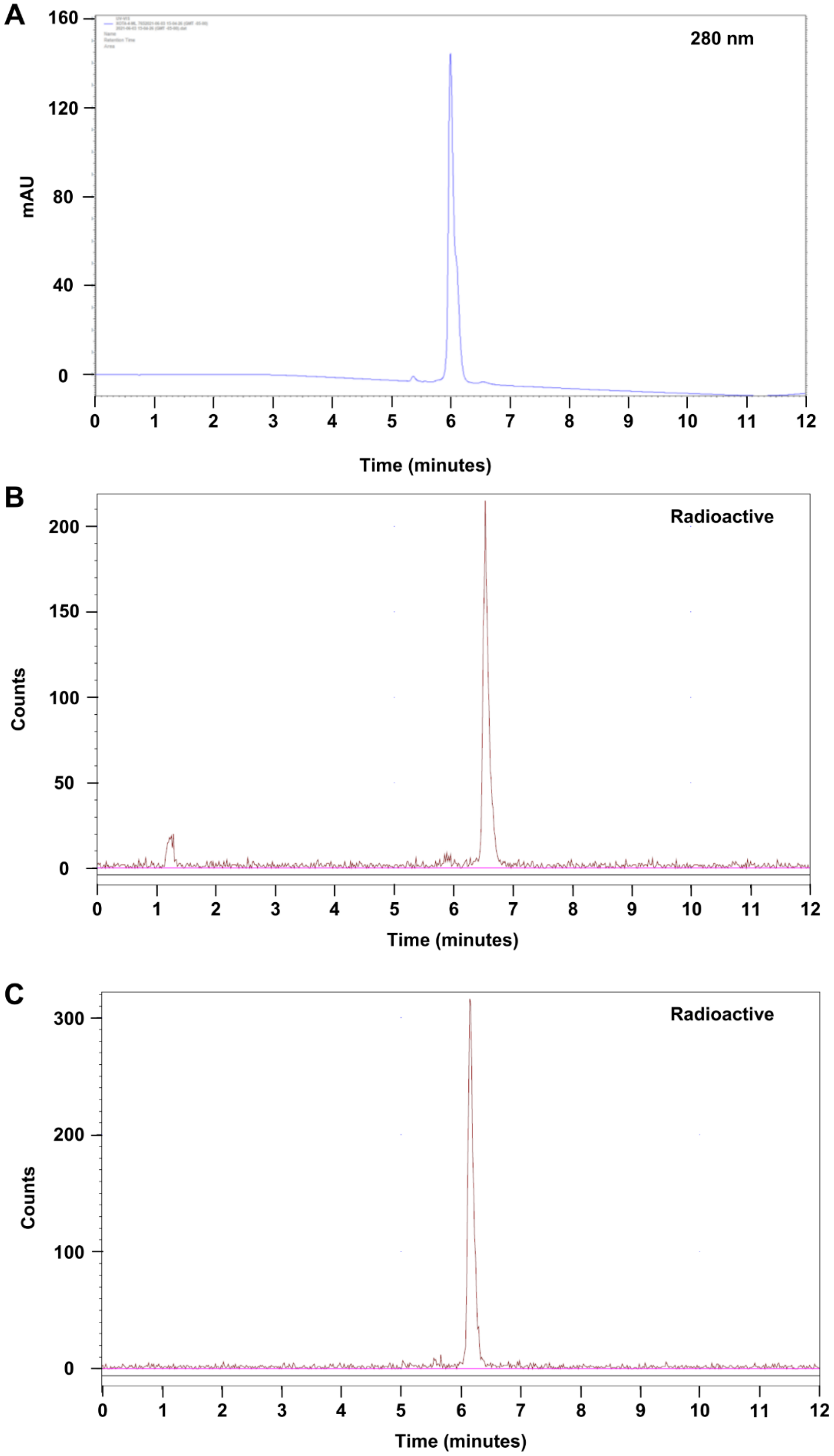

Supporting Figure 3. HPLC chromatograms showing the **(A)** absorbance of ^{67}Ga-MMC(FNIR-Tag)-TOC at 280 nm and the radioactive trace **(B)** before and **(C)** after purification.

A

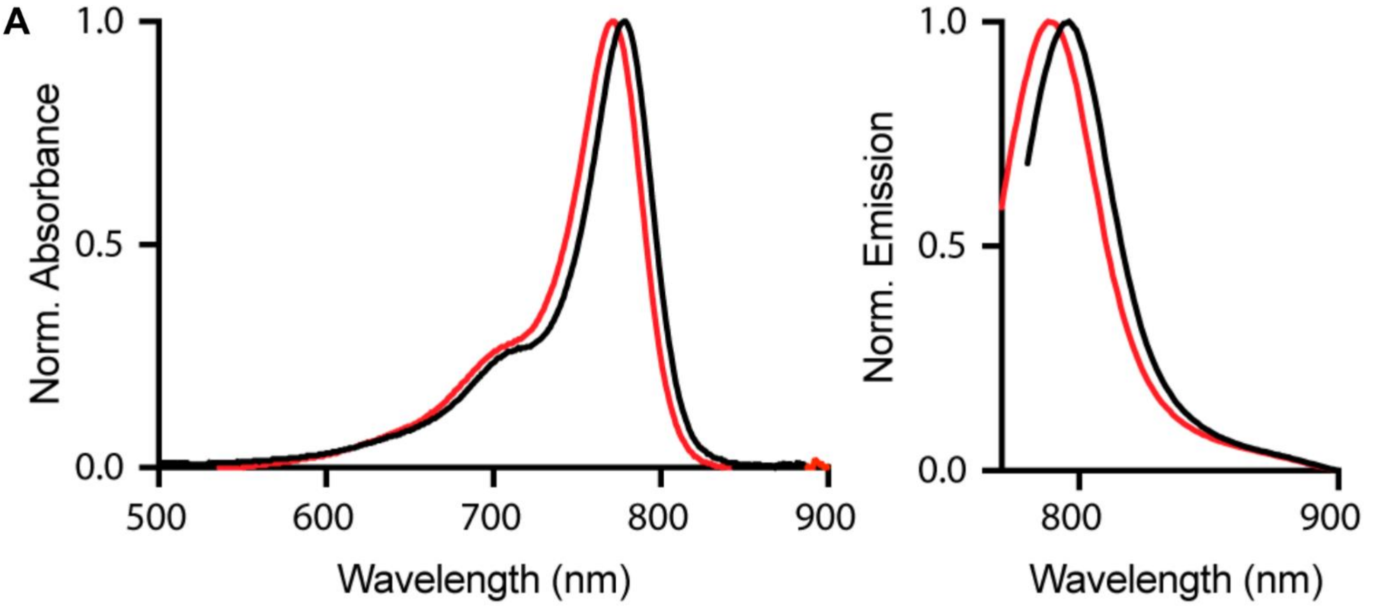

B

Compound	MW	Net Charge	cLogP	λ_{abs} (nm)	λ_{em} (nm)	φ_F
MMC(IR800)-TOC	2903.3	–5	0.0938	778	795	0.13
MMC(FNIR-Tag)-TOC	2894.5	–2	–6.68	772	788	0.13

Supplementary Figure 4. Spectral and physicochemical properties of fluorescent conjugates. (A) Normalized absorbance (left) and emission (right) spectra of MMC(IR800)-TOC (black trace) and MMC(FNIR-Tag)-TOC (red trace) in PBS. Φ_F values were obtained using an integrating sphere. **(B)** Summary of key physicochemical and optical properties of both conjugates.

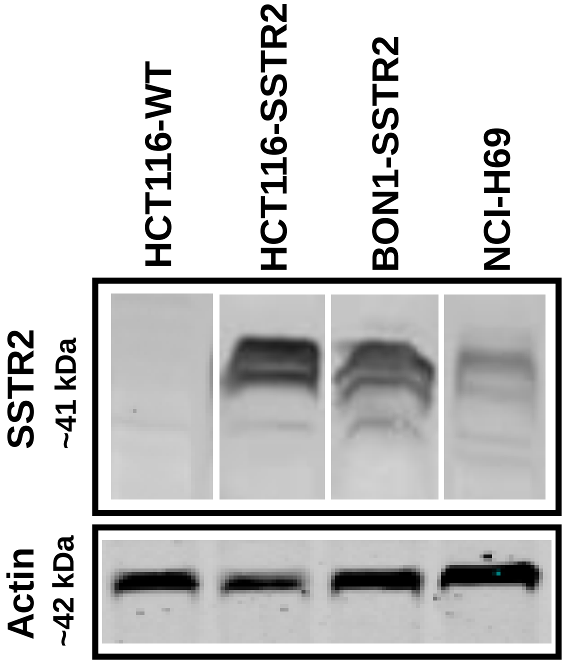

Supporting Figure 5. SSTR2 expression analysis using western blot. Cells were lysed in RIPA buffer and the whole cell lysate was used for western blot analysis using rabbit anti-SSTR2 (abcam-ab134152), and anti-actin (abcam-ab8227) primary antibodies, and IR800 goat anti-rabbit IgG (Li-COR) secondary antibody.

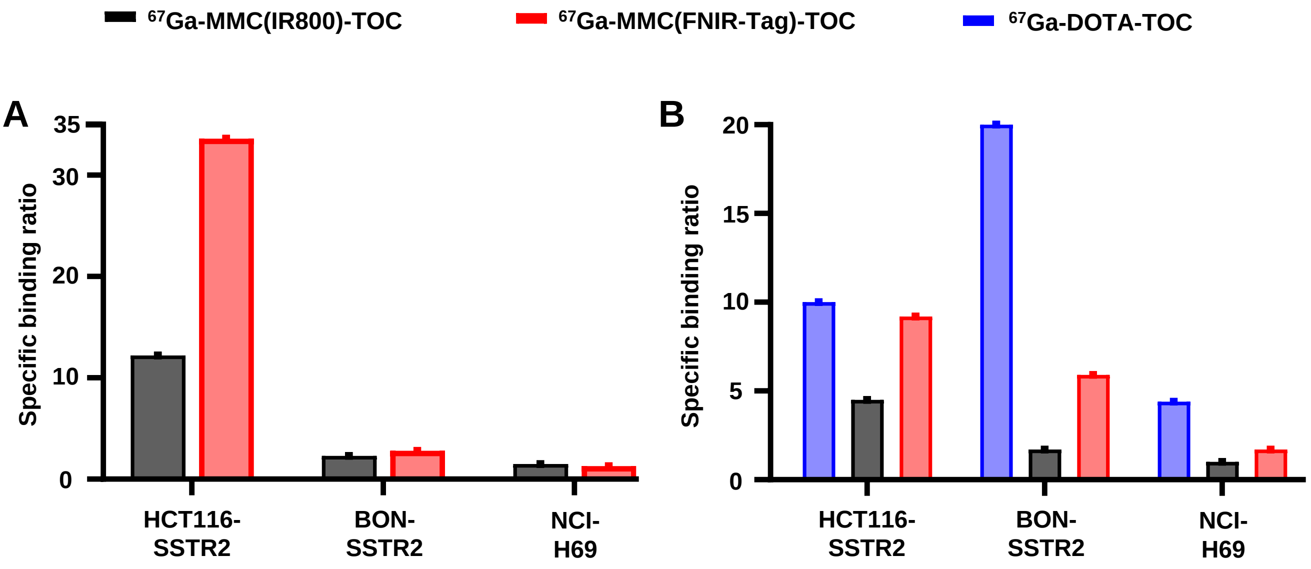

Supporting Figure 6. Specific binding ratios of dual-labeled conjugates *in vitro*. Analysis of blocked samples in **(A)** flow cytometry and **(B)** radioactive uptake shows higher specific binding ratios for FNIR-Tag compared with IR800.

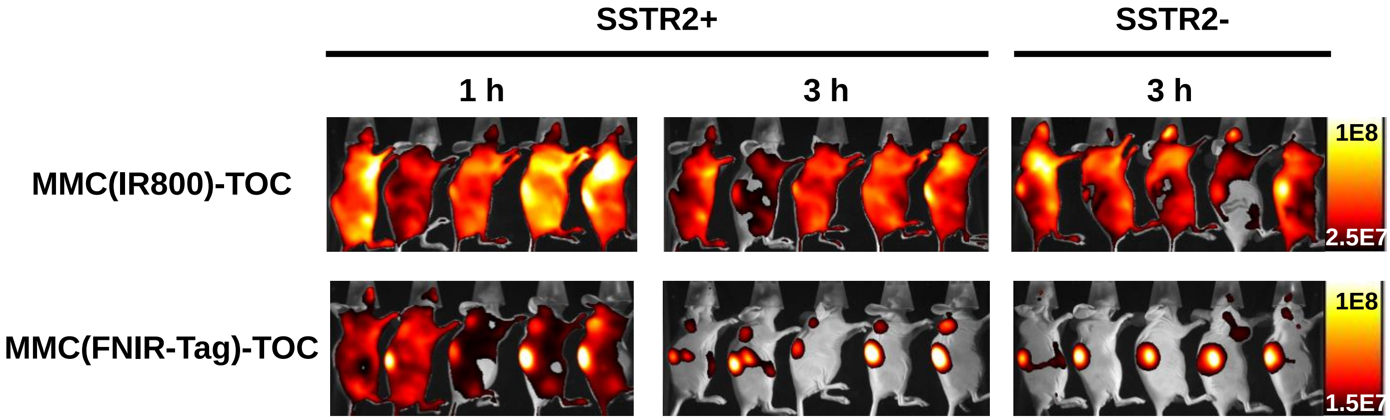

Supporting Figure 7. *In vivo* NIRF imaging of complete mouse cohorts injected with fluorescent conjugates shown at their optimal individual imaging scale. Comparison of mice with HCT116-SSTR2/WT (SSTR2+/-) xenografts after injection of 2 nmol MMC(IR800)-TOC or MMC(FNIR-Tag)-TOC at 1 h and 3 h (n = 5/group). Imaging scale, Photons/sec/mm^2.

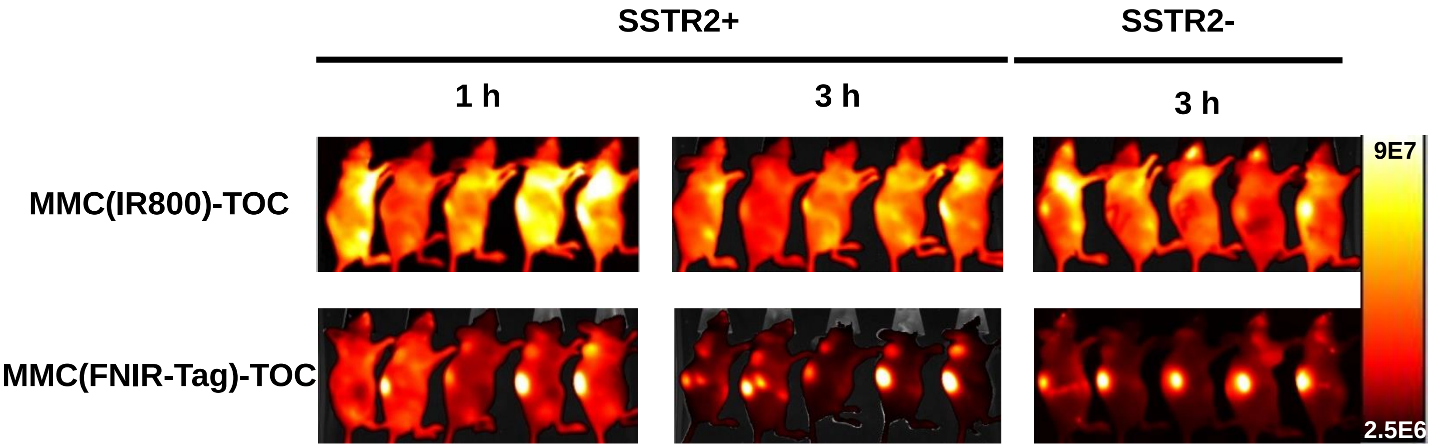

Supporting Figure 8. ***In vivo*** **NIRF imaging of complete mouse cohorts injected with fluorescent conjugates shown at the same imaging scale.** Reproduction of Supplementary Fig. 5 with mouse cohorts at the same imaging scale. Imaging scale, Photons/sec/mm^2.

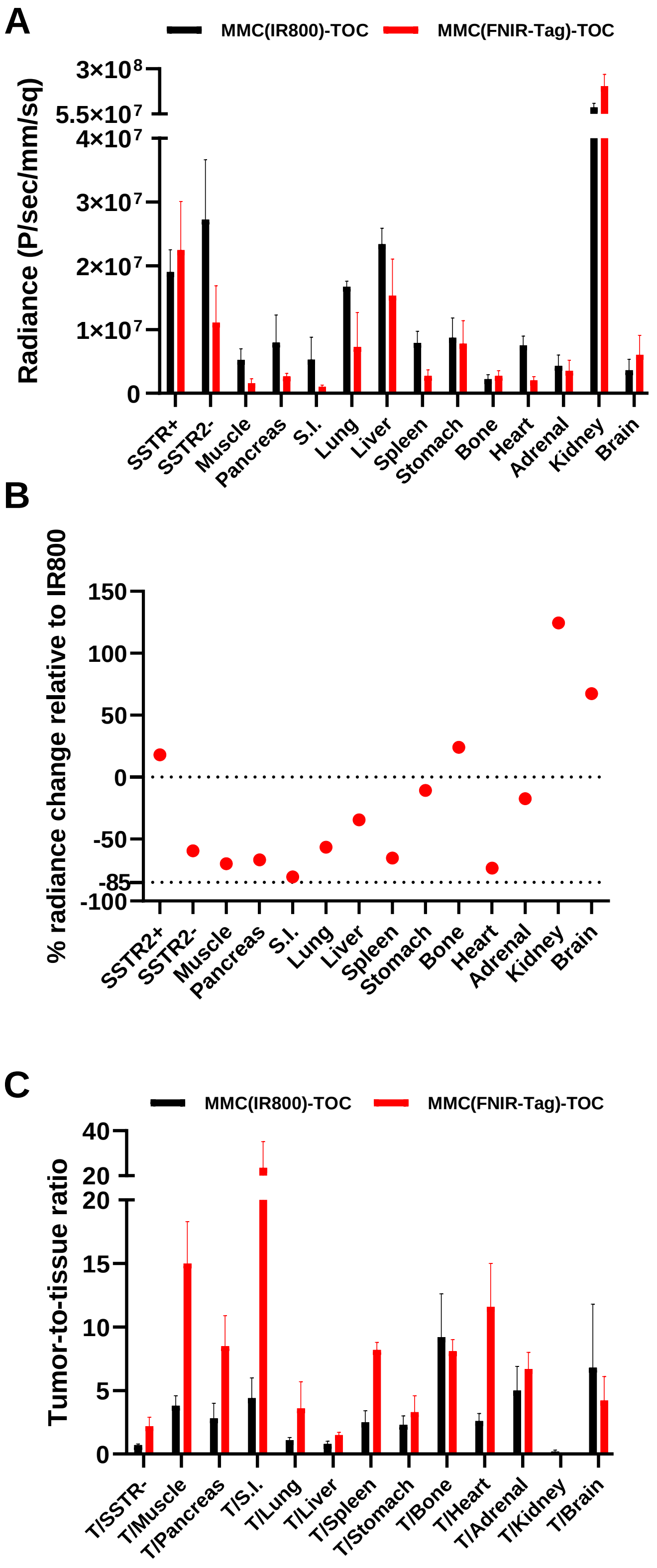

Supporting Figure 9. Complete *ex vivo* image analysis. Determination of tissue fluorescence from **(A)** *ex vivo* NIRF imaging, **(B)** % fluorescence change with FNIR-Tag relative to IR800, and **(C)** TBRs. Results are presented as mean ± s.d. (*n* = 4/group) except for **(B)**, which is shown as data on average.

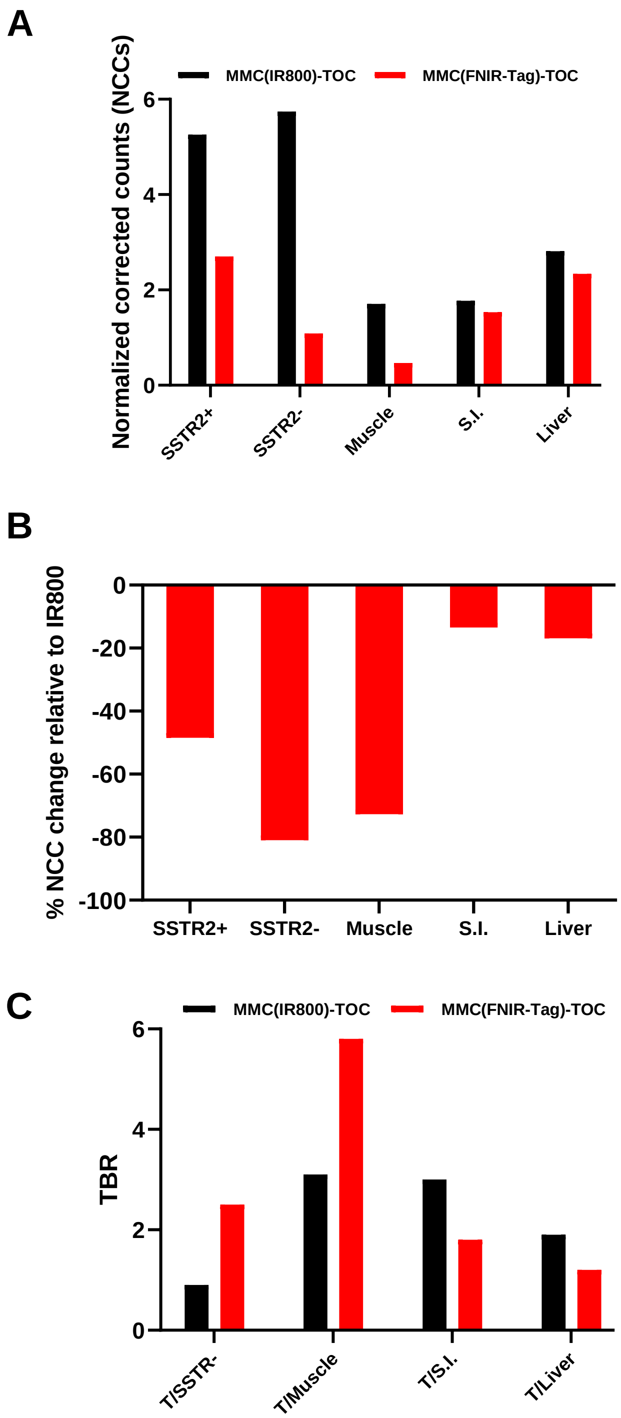

Supporting Figure 10. Image analysis of CFT. MMC(FNIR-Tag)-TOC and MMC(IR800)-TOC uptake in the HCT116-SSTR2/WT dual implant animal model as determined by **(A)** normalized corrected counts (NCCs), **(B)** % NCC change with FNIR-Tag relative to IR800, and **(C)** TBRs. Since 1 representative mouse was randomly selected from each group at the conclusion of the feasibility study (Fig. 4A, Supplementary Figs. 5 and 6), results are presented as mean only. The pancreas could not be accurately located in the CFT and was not analyzed.

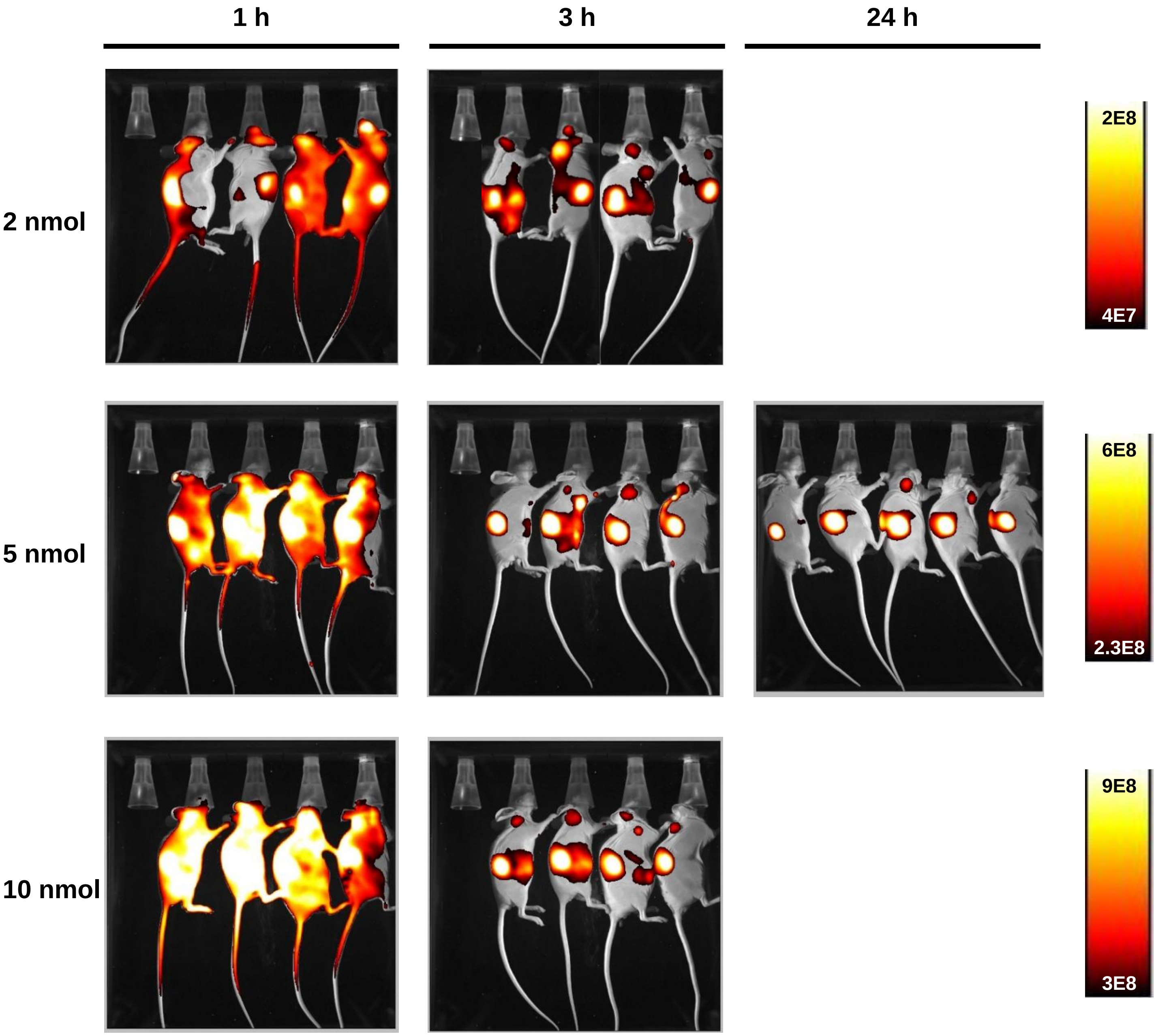

Supporting Figure 11. ***In vivo*** **NIRF imaging of complete mouse cohorts in the dose and time finding study.** NCI-H69 tumor-bearing mice (n = 4-5/group) were injected with increasing doses of ^{67}Ga-MMC(FNIR-Tag)-TOC and imaged at the indicated time points. Images shown at the optimal individual scale per dose.

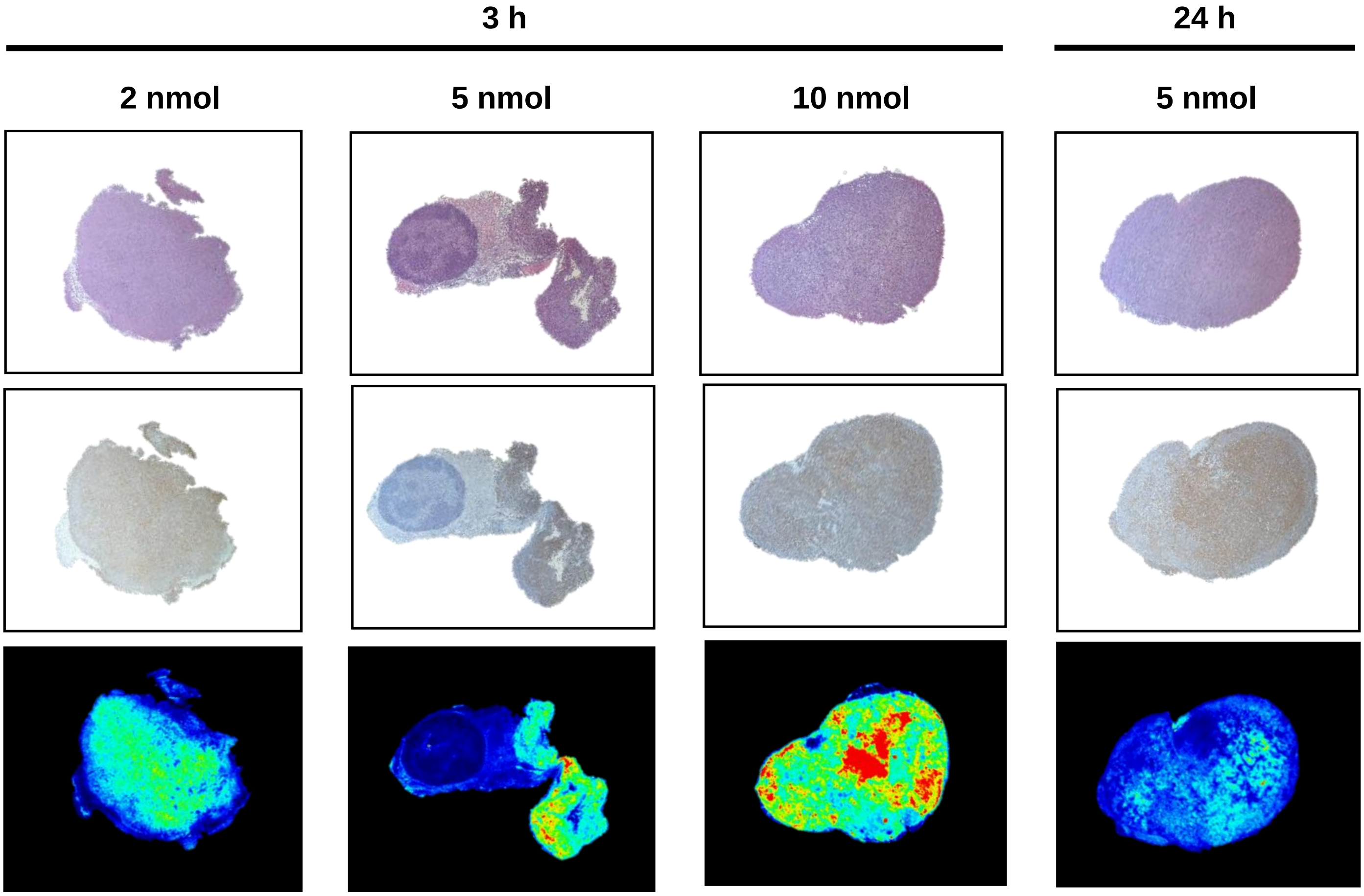

Supporting Figure 12. Qualitative correlation between SSTR2 expression and accumulation of MMC(FNIR-Tag)-TOC in the dose and time finding study. Mice with NCI-H69 tumors were injected with increasing doses of MMC(FNIR-Tag)-TOC and imaged at 3 h and 24 h p.i. Tumors were resected after imaging and cryosections were prepared. Histopathology and NIRF imaging revealed that, regardless of dose, fluorescence signal corresponded to areas with histologic evidence of tumor (confirmed by H&E) and co-localized with SSTR2 (confirmed by IHC).

Supporting Table 1. Radiance (Photons/sec/mm^2) for 2, 5 and 10 nmol of ^{67}Ga-MMC(FNIR-Tag)-TOC in NCI-H69 xenografts at 3 h and 24 h p.i. Results are presented as mean ± s.d. (*n* = 4-5/group). S.I., small intestine.

	**2 nmol**	**5 nmol**	**10 nmol**	**5 nmol-24h**
**Tumor**	1.1E8 ± 5.4E7	2.7E8 ± 9.7E7	3.0E8 ± 1.0E8	1.8 E8 ± 7.2E7
**Muscle**	8.6E6 ± 2.7E6	1.6E7 ± 6.7E6	3.2E7 ± 9.5E6	1.6E7 ± 2.8E6
**Pancreas**	3.2E7 ± 1.2E7	7.1E7 ± 2.6E7	8.6E7 ± 1.3E7	4.4E7 ± 1.4E7
**S. I.**	2.2E7 ± 6.8E6	2.3E7 ± 5.6E6	2.0E7 ± 1.5E7	3.4E7 ± 1.2E7

Supporting Table 2. Biodistribution (%IA/g) for 2, 5 and 10 nmol of ^{67}Ga-MMC(FNIR-Tag)-TOC in NCI-H69 xenografts at 3 h and 24 h p.i. Results are presented as mean ± s.d. (*n* = 4-5/group). S.I., small intestine.

	**2 nmol**	**5 nmol**	**10 nmol**	**5 nmol-24h**
**Tumor**	1.8 ± 0.9	1.9 ± 0.7	1.5 ± 0.5	0.8 ± 0.1
**Muscle**	0.1 ± 0.01	0.2 ± 0.1	0.2 ± 0.1	0.1 ± 0.02
**Pancreas**	0.9 ± 0.2	1.1 ± 0.3	0.8 ± 0.2	0.3 ± 0.1
**S. I.**	0.3 ± 0.1	0.5 ± 0.1	0.7 ± 0.1	0.2 ± 0.1
**Blood**	0.3 ± 0.1	0.5 ± 0.1	0.4 ± 0.1	0.1 ± 0.02
